# Hope for bone regeneration: The versatility of iron oxide nanoparticles

**DOI:** 10.3389/fbioe.2022.937803

**Published:** 2022-08-25

**Authors:** Nan Wang, Yimin Xie, Zhipeng Xi, Zehua Mi, Rongrong Deng, Xiyu Liu, Ran Kang, Xin Liu

**Affiliations:** ^1^ Affiliated Hospital of Integrated Traditional Chinese and Western Medicine, Nanjing University of Chinese Medicine, Nanjing, China; ^2^ Hospital for Skin Diseases, Institute of Dermatology Chinese Academy of Medical Sciences, Peking Union Medical College, Nanjing, China; ^3^ Department of Orthopedics, Nanjing Lishui Hospital of Traditional Chinese Medicine, Nanjing, China

**Keywords:** iron oxide nanoparticles, tissue engineering, bone regeneration, scaffolds, stem cells

## Abstract

**Abstract:** Although bone tissue has the ability to heal itself, beyond a certain point, bone defects cannot rebuild themselves, and the challenge is how to promote bone tissue regeneration. Iron oxide nanoparticles (IONPs) are a magnetic material because of their excellent properties, which enable them to play an active role in bone regeneration. This paper reviews the application of IONPs in bone tissue regeneration in recent years, and outlines the mechanisms of IONPs in bone tissue regeneration in detail based on the physicochemical properties, structural characteristics and safety of IONPs. In addition, a bibliometric approach has been used to analyze the hot spots and trends in the field in order to identify future directions. The results demonstrate that IONPs are increasingly being investigated in bone regeneration, from the initial use as magnetic resonance imaging (MRI) contrast agents to later drug delivery vehicles, cell labeling, and now in combination with stem cells (SCs) composite scaffolds. In conclusion, based on the current research and development trends, it is more inclined to be used in bone tissue engineering, scaffolds, and composite scaffolds.

## Introduction

Orthopedic diseases are the second leading cause of disability worldwide, as living conditions improve and people live longer, skeletal diseases are becoming more frequent, and their incidence is increasing every year (Rasker, 1995; Jakob et al., 2013; Weinstein, 2016). Among them, bone defects caused by trauma, tumors, chronic inflammation, infection, osteoporosis and congenital deformities caused great physical, and emotional damage to patients and diminished their quality of life (Gao et al., 2022; Li et al., 2017; Migliorini et al., 2021; Li et al., 2022b). Bone is a living organ that can heal and remodel itself. Nonetheless, extensive defects of bone and cartilage require the necessary interventions. For osteochondral defects that are not self-repairing, autologous or allogeneic osteochondral grafts are currently used as the primary means. However, this has the disadvantages of infection, immune rejection, inflammation, and disease transmission, and these limitations have severely restricted their widespread use in clinical practice (Tang et al., 1998; Kowalczewski and Saul, 2018; Zhang et al., 2019c; Yao et al., 2019; Koons et al., 2020; Xu et al., 2021). Particularly for those with serious irregularities in bone and cartilage, their rational repair and reconstruction can be interesting and challenging (Fazzalari, 2011; Tevlin et al., 2014; Ho-Shui-Ling et al., 2018).

In recent years, magnetic nanoparticles (MNPs) have been widely used in medical research, such as magnetic resonance imaging (MRI), cell labeling, targeted drug delivery, antitumor, thermal therapy, and biosensing (Ferreira et al., 2016). Compared with other materials, MNPs have unique advantages, including low production cost, good biocompatibility, stable physicochemical properties, and low toxicity (Ozbey et al., 2015), and consequently, they are receiving increasing attention in the biomedical field. The magnetic materials currently most used in the medical field are based on iron oxide (IO), such as ferric oxide (Fe_2_O_3_) and ferroferric oxide (Fe_3_O_4_) (Ramimoghadam et al., 2014; Shokrollahi, 2017). Iron is an essential trace element in the body, and exogenous IO can be metabolized and degraded through various pathways when it enters the body, and it is relatively non-toxic (Xia et al., 2019c; Liu et al., 2020a). Because IO is more common in nature, easy to synthesise and particularly sensitive to magnetic fields, it has been researched more frequently (Paik et al., 2015; Salehiabar et al., 2018). And mainly due to the mobility in response to magnetic fields (Kitture and Ghosh, 2019), iron oxide nanoparticles (IONPs) have been approved by the FDA and are the most widely studied material in the field of nanomedicine (Bobo et al., 2016). However, due to the easy aggregation of bare iron and the potential toxicity caused by the release of iron ions, most of them are coated with biocompatible ligands, which can affect cell proliferation, adhesion, migration, differentiation, movement, and distribution under the action of an external magnetic field (Vangijzegem et al., 2019). IONPs can be visualized by MRI and can also promote cell differentiation, inhibit osteoclast formation, and enhance osteogenesis (Li et al., 2018a; Xia et al., 2018b; Liu et al., 2018). Overall, IONPs have a strong ability to promote bone regeneration, good biocompatibility and chemical stability, as well as no significant intrinsic toxicity, which is superior to other magnetic nanoparticles, and are widely used in bone regeneration therapy (Paik et al., 2015; Li et al., 2018a; Xia et al., 2018b; Salehiabar et al., 2018).

**FIGURE 1:**
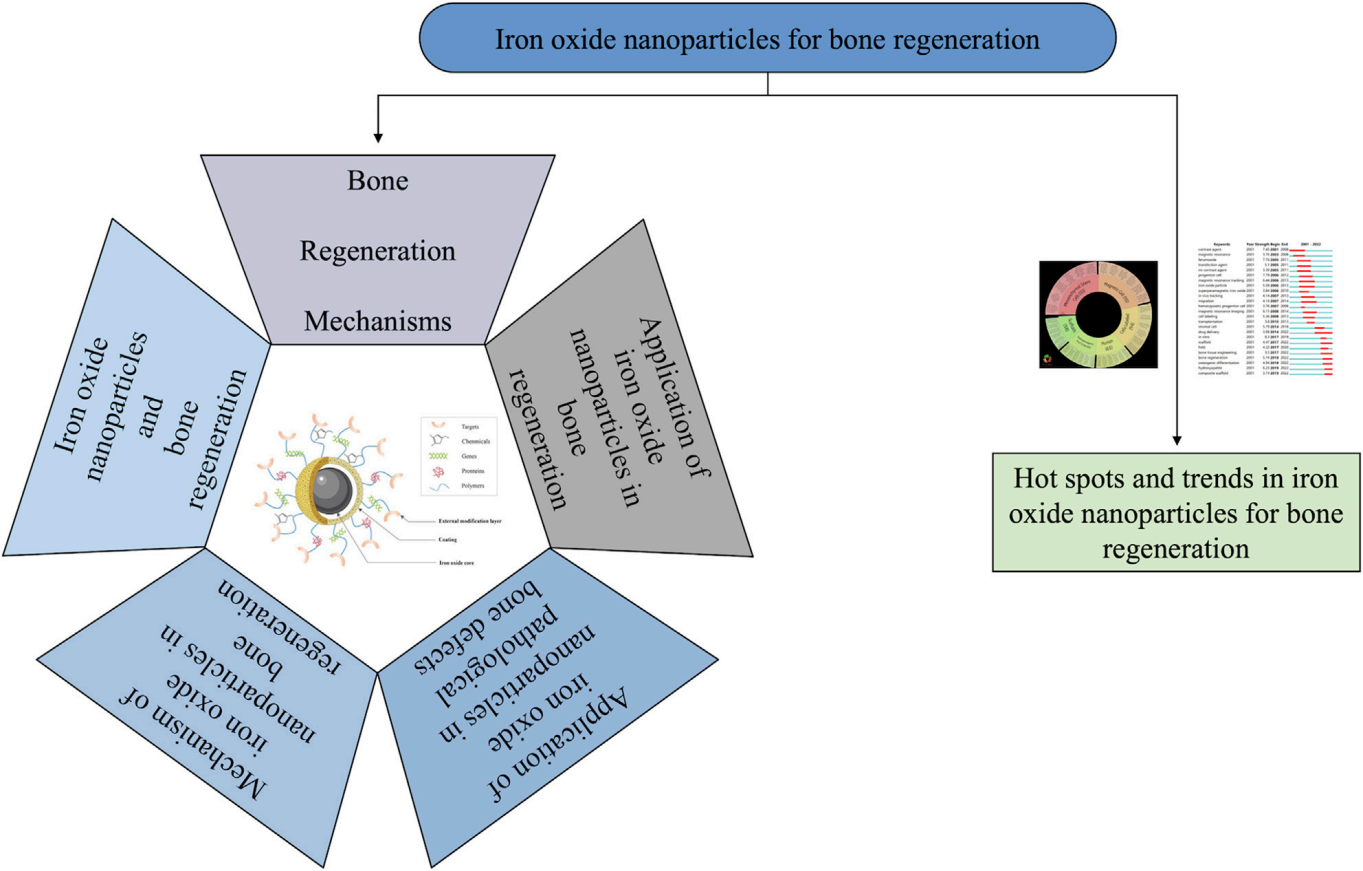
General diagram of the core content of the article. This paper contains six main contents, which are respectively bone regeneration mechanisms, iron oxide nanoparticles and bone regeneration, application of iron oxide nanoparticles in bone regeneration, application of iron oxide nanoparticles in pathological bone defects, mechanism of iron oxide nanoparticles in bone regeneration, and hot spots and trends in iron oxide nanoparticles for bone regeneration.

Consequently, this article summarizes some of the achievements and progress in the use of IONPs in bone regeneration in recent years and provides a summary and perspective of the research hotspots and trends. Specifically, the characteristics of IONPs and their application in bone repair, mechanism studies, and safety were discussed. Furthermore, this article adopts a bibliometric approach to analyze and discuss the hot spots and research trends. Through the above research, it is hoped that this contribution will guide the study of bone regeneration (Figure 1).

## Bone regeneration mechanisms

**FIGURE 2:**
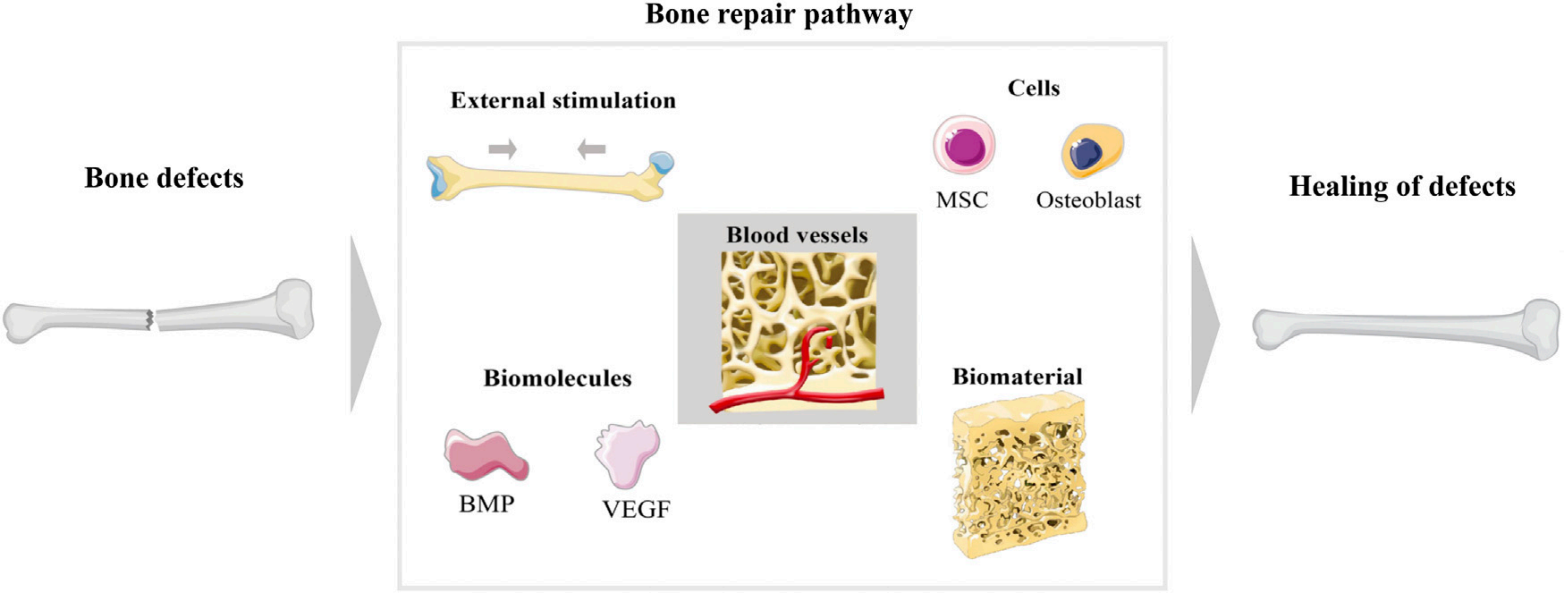
Main schematic diagram of bone regeneration. There are five ways to promote bone repair after bone injury, which are external stimulation, cells, blood vessels, biomolecules, and biomaterials.

The skeleton is a vital organ that supports the body and remains under stress and weight for the whole life (Zhu et al., 2021). Usually, bone resorption and destruction is a dynamically balanced metabolic process. In the micro-ecological environment of bone, various cells are involved, including a large number of osteocytes, osteoclasts, and immune cells (Mohammadi et al., 2018). Osteocytes and osteoblasts play a key role in bone formation, while osteoclasts are involved in the resorption and regeneration of bone tissue (Torgbo and Sukyai, 2018). In addition, type I collagen, the inorganic minerals calcium (Ca^2+^), and phosphate (PO_4_^3-^) are still important components of bone structure (Jang et al., 2009). Physiologically, it has a certain degree of self-healing when minor bone defects or bone destruction occurs; however, this ability is limited, especially in the case of trauma, infections, and tumours that result in bone defects worth more than 2 cm (Yang et al., 2018; Zhang W. et al., 2019a; Wagner et al., 2019). More importantly, bone defects can also lead to bone ischaemia, bone loss, and osteonecrosis, which further lead to a failure of bone healing (Loi et al., 2016). When a bone defect cannot heal itself, additional means are essential to achieve the goal of stimulating bone regeneration. How to maximize the optimal repair of bone defects has been a hot topic. The current methods used for bone regeneration consist of five main components depending on the pathogenesis of the bone injury (Figure 2): 1) external stimulation, 2) cells, 3) blood vessels, 4) biomolecules, and 5) biomaterials (Saiz et al., 2013; Hankenson et al., 2015; Lopes et al., 2018). In current studies, several methods are used in combination to enhance the effectiveness of the therapy. External stimulation such as magnetic fields promote bone regeneration (Singh et al., 2014; Zhuang et al., 2018); cellular therapy is currently focused on the use of MSCs (Jia et al., 2019); vascular regeneration is essential to ensure cellular metabolism and nutrition (Xia et al., 2018b); biomolecules include growth factors and bone morphogenetic proteins (Yu et al., 2020); and biomaterials are currently an important modality in the treatment of bone defects, and the development of various scaffolds has extended the effect of osteogenesis. For the study of scaffolds, various requirements need to be met, of which biocompatibility, absorbability, mechanical properties, non-toxic side effects, and bubble structure are the most important, as shown in Table 1.

**TABLE 1:** Requirements for biomaterial scaffolds in bone regeneration.

Requirements	Purpose	References
Biocompatibility	Supports cellular activity and molecular signalling without causing immune rejection or inflammatory responses	Murphy et al. (2010); Chen et al. (2022)
Absorbability	Provides space for new tissue to grow inwards; allows for controlled degradation *in vivo*	Williams, (2008)
Mechanical properties	Meets mechanical strength needs and provides transfer properties	Yi et al. (2016)
non-toxic side effects	The various properties should be carried out under non-toxic conditions	Mondschein et al. (2017)
Bubble structural	Can provide a reasonable amount of space for bone regeneration	Hou et al. (2019)

In response to the mechanisms of bone regeneration, current research focuses on promoting bone healing through the modification of external conditions. There are various methods of bone regeneration, but in order to enhance the effect of bone growth, a combination of methods is usually applied, and this is most noticeable in the case of biological scaffolds. If biomolecules, cells, and IONPs are mixed in a scaffold, it retains all the advantages of a scaffold but also has molecular, cellular, and nanoparticle-related characteristics (Ou et al., 2019; Yang et al., 2019; Petretta et al., 2021). Although some results have been reached, the search for the design of scaffolds with ideal loading molecules and cells with good biological effects, suitable mechanical properties, optimized bubble structures with controlled degradability, and non-toxic to tissues are still the direction being pursued.

## Iron oxide nanoparticles and bone regeneration

### Overview of iron oxide nanoparticles

**FIGURE 3:**
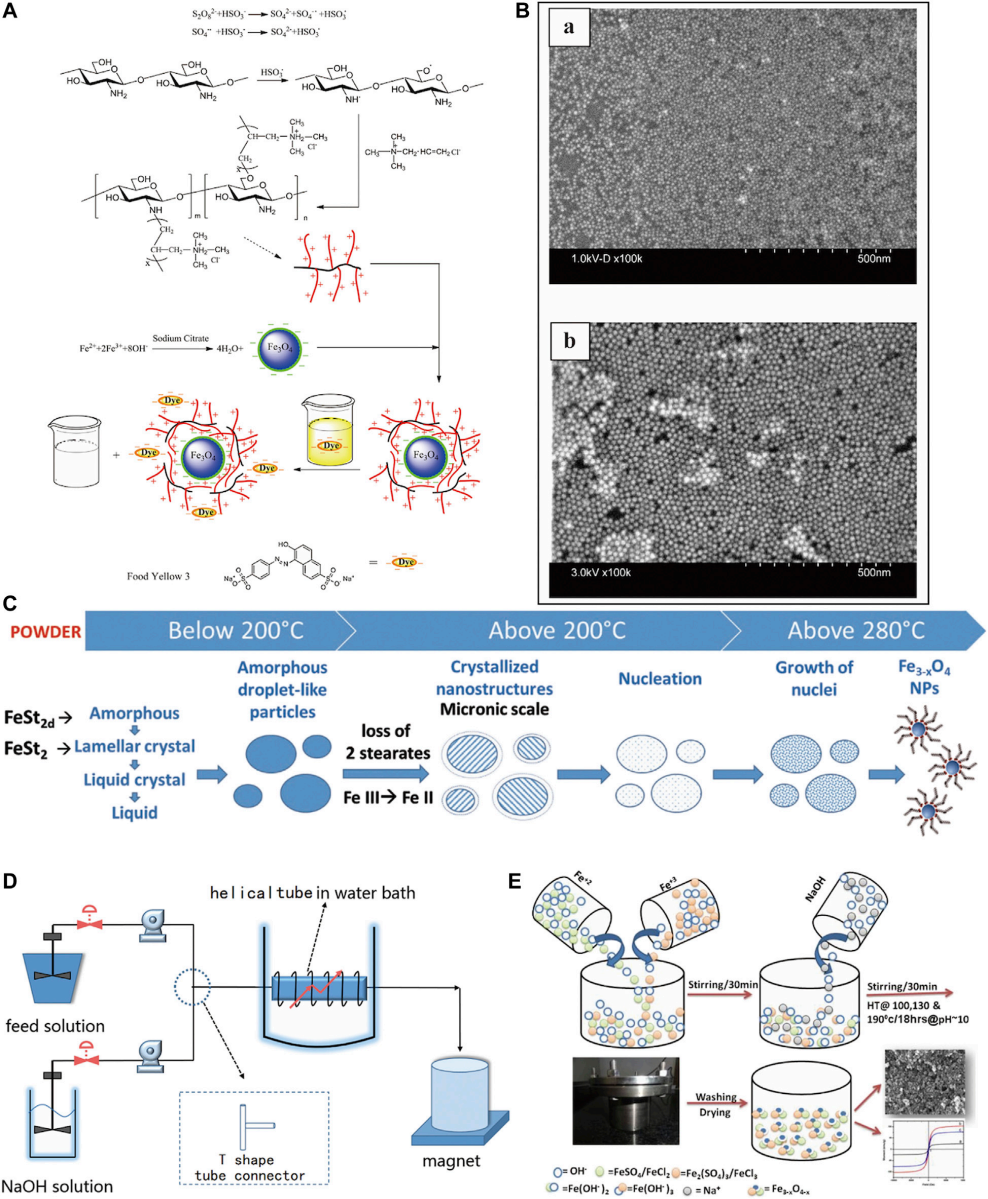
Methods for the preparation of IONPs. **(A)** Wet method preparation process of CTS-g-PTMAAC/SC-Fe_3_O_4_ composite magnetic nanoparticles (Chen et al., 2016); **(B)** Images of IONPs prepared by immunoprecipitation technique under scanning electron microscope (SEM), **(a)** 5 nm, **(b)** 20 nm (Mengesha et al., 2022); **(C)** IONPs obtained by thermal decomposition (Cotin et al., 2022); **(D)** Preparation of IONPs by co-precipitation method in microchannel reactor (Lin et al., 2017); **(E)** Preparation of high saturation magnetic IO nanomaterials (Bhavani et al., 2017).

Among the various nanoparticles, magnetic materials are more attractive to researchers because of their unique responsiveness to magnetic fields (Dhivya et al., 2015). Currently, magnetic materials commonly used in the biomedical field include iron, cobalt, titanium, and nickel metal alloys as well as IO, ferrite (BaFe_12_O_19_, CoFe_2_O_4_), and nano-magnetic hydroxyapatite, among which magnetic IONPs are the most widely studied because iron is an essential element for the human body and can be sensitive to magnetic fields (Li et al., 2016). It can be combined with growth factors, stem cells (SCs), drugs, and other biologics so that be labeled, imaged, thermally treated, and disease targeted (Ghosh et al., 2015; Accomasso et al., 2016; Rajan et al., 2020). The biomedical applications of IONPs are mainly based on their superparamagnetic properties. Superparamagnetic iron oxide nanoparticles (SPIONs) are a type of IONPs with a diameter of 10–100 nm and can generate strong magnetism in a weak magnetic field, and its magnetism can disappear with the withdrawal of the external magnetic field (Laurent et al., 2008; Kircher et al., 2011). Based on its superparamagnetic properties, IONPs are usually used in MRI examinations and targeted to specific areas under the action of an external magnetic field to achieve targeted therapeutic effects (Radeloff et al., 2020). Maria et al. (Volokhova et al. 2022) made SiO_2_-coated magnetic iron oxide nanoparticles into α-Fe@SiO_2_ with a cubic morphology, which exhibited higher MRI relaxation than iron oxide alone and could show high-performance contrast effects. Zheng et al. (2022) found that allantoin phosphate can deliver iron oxide nanoparticles precisely to bone tissue for targeted synergistic treatment of osteoporosis. At present, the preparation of IONPs primarily consists of dry and wet methods (Yu et al., 2016; Huang et al., 2018) (Figure 3). The wet methods are more commonly used and include thermal decomposition, chemical co-precipitation, sol-gel, ball milling and atomic layer deposition (Cotin et al., 2022; Kiyani et al., 2022). The co-precipitation method is more commonly used in the synthesis of MNPs and allows for better control of the size and magnetic properties of the material, but the MNPs have a tendency to aggregate due to the small particle size (Lin et al., 2017). The high-temperature pyrolysis method is mainly used to synthesis ultra-fine powders and crystals of different materials, but it cannot obtain NPs smaller than 10 nm in size (Bhavani et al., 2017). Therefore, different extraction methods have their advantages and disadvantages, and researchers should select the appropriate method for generating IONPs according to the specific situation.

### Iron oxide nanoparticle physicochemical properties

**FIGURE 4:**
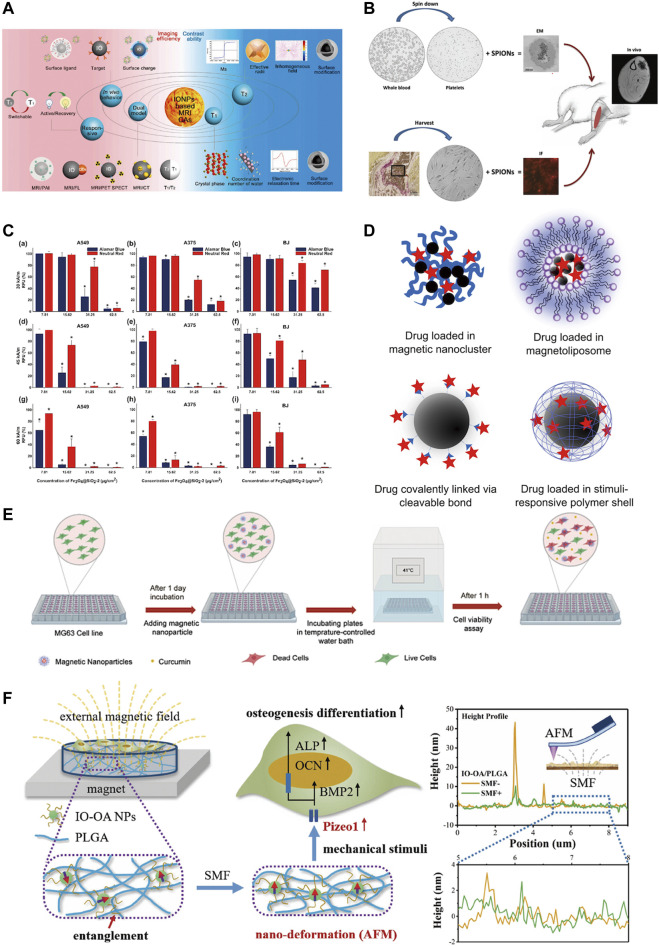
Physicochemical properties of iron oxide nanoparticles. **(A)** IONPs are used for MRI examination due to their good magnetic properties and biocompatibility (Zhao et al., 2022); **(B)** IONPs are readily absorbed by cells and can label cells for cell tracking (Iyer et al., 2017); **(C)** IONPs have magnetic heating properties and can substantially promote tumor cell death *in vitro* (Iacoviță et al., 2021); **(D)** IONPs are used for targeted magnetic drug delivery (Hola et al., 2015); **(E)** CSLM images showing MG-63 cancer cell line stained alive/dead at 41°C (Khodaei et al., 2022); **(F)** IO-OA/PLGA produces mechanical stimulation in the presence of an external magnetic field and promotes osteogenic differentiation of MC3T3-E1 cells (Hao et al., 2019).

The physicochemical properties of IONPs include magnetic properties, chemical stability, modifiability, biocompatibility and ease of surface functionalization (Clark et al., 2005) (Figure 4). In particular, the magnetic properties make it multifunctional. IONPs have long been used for MRI contrast for its uniquely magnetic and highly biocompatible (Zhao et al., 2022). Its good biocompatibility allows it to label specific cells without causing damage (Moise et al., 2017). With their biocompatible, magnetic, small size and customised surface coating properties, SPIONs can be used for the delivery and monitoring of small molecules, drugs and cells, particularly in areas of muscle, bone or cartilage (Iyer et al., 2017).

Magnetism is the most important property of IONPs. SPION can sparingly deliver drugs to specific areas under the action of an external magnetic field and can undergo magnetothermal transformation to kill tumour cells which ultimately treats tumours (Iacoviță et al., 2021; Pucci et al., 2022). Additionally, magnetic materials can have mechanical properties that can regulate cell behaviour and promote bone regeneration (Hao et al., 2019). The small particle size of IONPs allow them to overcome the biological barrier for wide distribution in the body, and they can also be excreted from the body by themselves (Hola et al., 2015). Studies have indicated that very small particles with hydrodynamic diameters up to 5 nm–8 nm can be excreted from the kidneys; while larger IONPs are readily captured by the reticuloendothelial system (RES), allowing imaging of the liver and spleen and detection of areas of inflammation; it ranging in size from about 20 to 150 nm may also accumulate in the stomach, bones and kidneys (Dadfar et al., 2019). Thus, the particle nature of the iron oxide nanoparticles is also an important characteristic to ensure its functionality.

### Iron oxide nanoparticle structure

**FIGURE 5:**
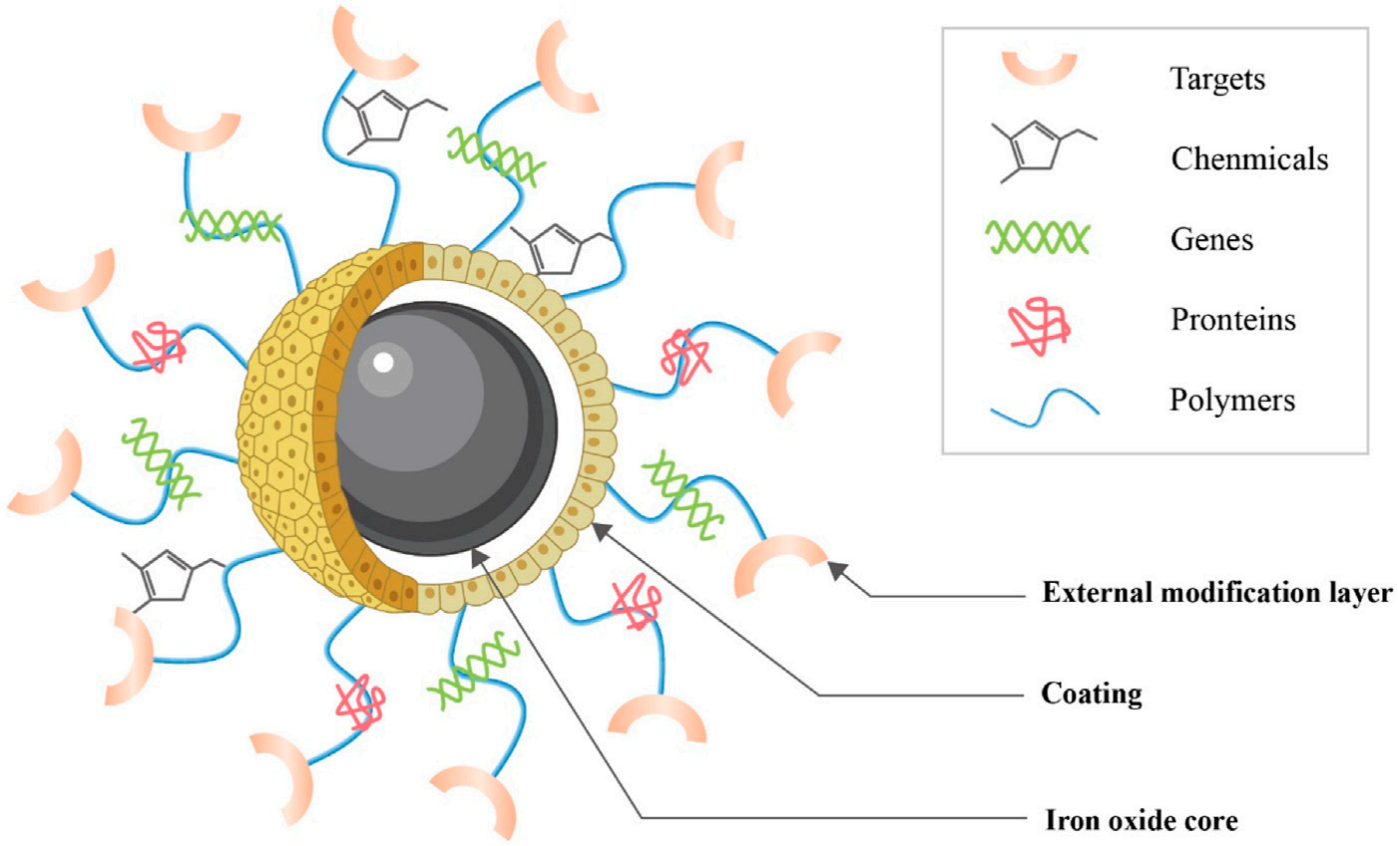
Structure of IONPs. IONPs consist primarily of an IO core, a coating and an external modification layer (This diagram is drawn from (Dasari et al., 2022)).

IONPs consist primarily of an IO core, a coating, and an external modification layer (Figure 5). The core is a large amount of IO, which ensures that it has a certain magnetism. Uncovered IO is toxic because it tends to collect and react chemically, so an organic or inorganic coating needs to be applied to the surface of the core, which reduces IO oxidation, toxicity, and enhances its biocompatibility (Dadfar et al., 2019). Current coating materials include organic, inorganic, and organic-inorganic composites (Ma et al., 2013). Organic coatings include mainly dextran, chitosan, citrate, agarose, collagen, polylactic acid (PLA), polycaprolactone (PCL), polyglycolic acid (PGA), and polylactic acid-glycolic acid copolymer (PLGA) (Iyer et al., 2017; Marin et al., 2020); the inorganic coating consists mainly of silica, calcium phosphate, calcium silicate, and calcium phosphate cement complexes (Ferrage et al., 2017); organic-inorganic composites such as mineralized collagen, which retain the common properties of both while giving the scaffold good rigidity and toughness (Li et al., 2021b). The main purpose of the coating is generally to restrict particle aggregation and provide hydrophilicity, stability, and biocompatibility to the internal core (Accomasso et al., 2016; Harrison et al., 2017). In order to bind specifically to the receptor, molecular modifications can be applied outside the coating, or the therapeutic agent can be loaded so that the combination can be used for specific targeted therapy (Li et al., 2012).

### Iron oxide nanoparticle safety

**FIGURE 6:**
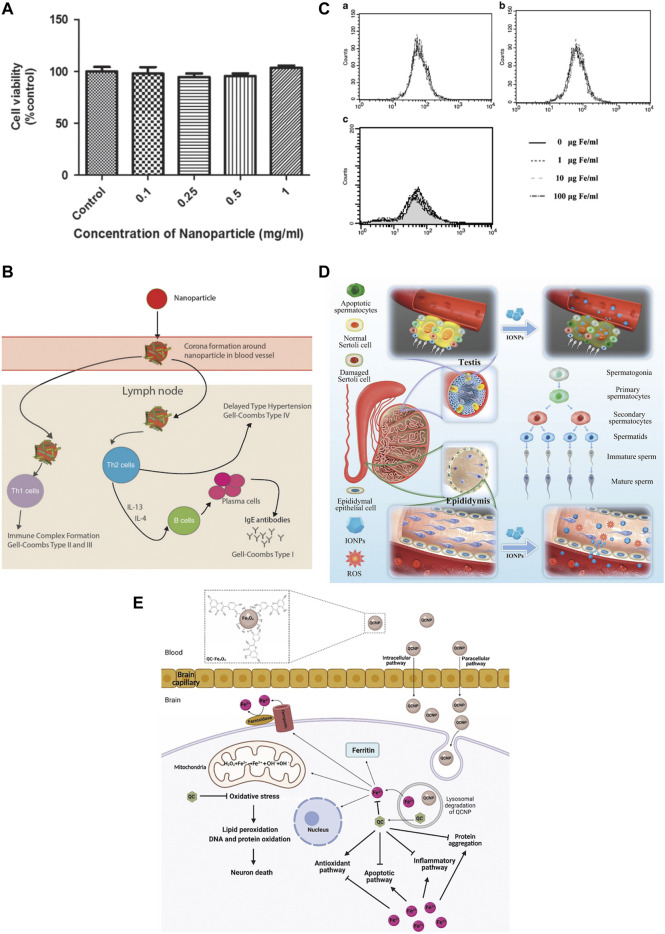
Safety of IONPs. **(A)** MTS assay showed that different concentrations of Au-Fe3O4 nanoparticles were not significantly toxic to CHO cells (Pariti et al., 2014); **(B)** USPIO could induce an immune response (Daldrup-Link, 2017); **(C)** Flow cytometry assay showed that SPIO was incubated directly on hMSCs for a long time (72 h) and had no significant effect on ROS(Yang et al., 2011); **(D)** IONPs can cause reversible damage to the reproductive system of male mice without affecting major organs (Yang et al., 2022); **(E)** Quercetin attenuates the neurotoxicity induced by IONPs(Bardestani et al., 2021).

One of the first things to ensure when IONPs are used for bone regeneration is safety (Figure 6). Studies have indicated that IONPs have superior biocompatibility and stability without significant intrinsic toxicity (Salehiabar et al., 2018). Most studies have demonstrated that low doses of IONPs are non-toxic to organisms (Pariti et al., 2014). In general, the toxicity of nanoparticles depends on their physical and chemical properties, such as particle size, surface properties, and chemical composition (Huang et al., 2017b). Therefore, before evaluating the toxicity of IONPs *in vitro* and *in vivo*, their physicochemical properties must be clarified. Various nanoparticles exhibit size-dependent toxicity *in vitro* and *in vivo* (Wu et al., 2022a). Under normal conditions, nanoparticles smaller than 10 nm are eliminated by the kidneys, while nanoparticles larger than 200 nm are phagocytosed (Rajan et al., 2020). The majority of IONPs are engulfed by hepatocytes, which on the one hand, facilitates their usefulness in liver imaging and, on the other hand, is a potential hazard to the body (Daldrup-Link, 2017). Although IONPs can stay in the body for a long time, they are biodegradable, can be metabolised from the circulation through a variety of pathways (Ventola, 2012). However, some IONPs are still non-degradable and it is not known whether there are serious effects on the body. It is also considered that IONPs can induce some toxic side effects in the body. IONPs have been found to cause significant toxicity in rats when kept in the body for long periods, causing reduced body weight, significant liver damage, and mild splenic toxicity (Verma et al., 2021). The interaction of the surface charge of IONPs between nanoparticles and biological components also substantially affects their toxic response (Woo et al., 2021). Toxic reactions are mainly manifested by impaired mitochondrial activity, membrane leakage, and morphological changes that negatively affect cell viability, proliferation, and metabolic activity, significantly weakening its therapeutic efficiency (Yang et al., 2011). Nanoparticle-treated cells may trigger immune-inflammatory reactions and other side effects if they migrate in tissues or accumulate at specific sites over time (Mahmoudi et al., 2010; Mahmoudi et al., 2012). IONPs were discovered to cause reversible damage to the reproductive system of male mice without affecting major organs (Yang et al., 2022). There are some solutions to these risks. For example, IONPs may damage neural tissue through free iron accumulation, protein aggregation, and oxidative stress, but quercetin may reduce neurotoxicity in clinical applications (Bardestani et al., 2021). The effect of IONPs on the inflammatory response is size-dependent, with smaller IONPs weakening the inflammatory response; this inflammatory response can also be attenuated by blocking actin polymerization, endoplasmic reticulum (ER) stress, or oxidative stress (Ying et al., 2022). New predictive models have also been developed as a method of toxicity screening to provide a safety profile for the clinical use of IONPs. Coccini et al. (2019) have developed a model for assessing human stem cells-based Fe_3_O_4_ NP toxicity screening and support using this approach to improve the safety of NPs to predict health outcomes with confidence. Hence, when utilizing IONPs, it is essential to understand their physicochemical properties. IO itself is toxic, but by choosing a suitable surface coating, reducing the dosage, reducing the diameter of the particles, and avoiding long-term aggregation in the same location, the toxic reaction can be effectively reduced. Measures should be taken in advance for different side effects, and extracts of some herbs can be used in synergy with IONPs to reduce toxicity. Models for predicting toxicity and safety assessment are also necessary for the rational use of nanoparticles.

### Effect of iron oxide nanoparticles on bone regeneration

IONPs, as a special magnetic particle, have a multifaceted effect on bone regeneration. Its function of promoting bone regeneration is closely related to its physicochemical properties. Among them, magnetism is an important prerequisite for the action of IONPs on bone regeneration. It has been demonstrated that IONPs not only respond to external magnetic fields, but also act as a magnetic domain themselves on the nanoscale, with nanoparticles forming magnetic fields to generate a number of biochemical reactions (Xie et al., 2019). When an external magnetic field is applied to bone defects, it promotes fracture healing and tissue repair, even without the intervention of IONPs(Shan et al., 2013; Thrivikraman et al., 2014). When IONPs are used to treat bone defects, a magnetic field is applied externally, which further enhances their magnetic effect, and this magnetism stimulates osteoblast-associated cells, activating the osteogenic pathway and exerting a bone regeneration effect (Jia et al., 2019). In addition, mechanical stimulation was found to promote osteogenic differentiation of stem cells (Meng et al., 2021). IONPs are mechanically stimulated by the effect of a magnetic field, which acts on the cells to generate internal biochemical signals and enhance bone regeneration (Wang et al., 2016; Zhuang et al., 2018). Furthermore, IONPs can generate magnetothermal conversion with the intervention of an alternating magnetic field (AMF), which is delivered to deep lesions to generate heat for the treatment of bone tumours and post-operative bone defects (Guo et al., 2017; Lee et al., 2018; Albarqi et al., 2019). The production of blood vessels is still essential for the regeneration of bone tissue. IONPs were identified to improve angiogenic properties and promote the formation of new blood vessels in bone defects, which was present in the presence or absence of static magnetic field (SMF) intervention (Singh et al., 2014; Xia et al., 2018b; Hu et al., 2018). It can be absorbed by cells through endocytosis (Kolosnjaj-Tabi et al., 2013), so it is used for cell tracking and targeting (Zhang and Zhang, 2005; Shen et al., 2013). This can detect the location of cells in bone regeneration and migration of SCs(Liao et al., 2022). Cells labelled with these nanoparticles are non-toxic and easily reach the desired effect. Note that for those IONPs that are smaller in size and have a longer half-life in the blood stream, it has significant bone targeting properties (Arami et al., 2015). In particular, it can remove reactive oxygen species from the body to regulate bone metabolism and improve post-menopausal bone loss (Yu et al., 2020). It has also been found that IONPs can inhibit osteoclast production and differentiation (Zheng et al., 2022). Its multifaceted action promotes the occurrence of bone regeneration.

## Application of iron oxide nanoparticles in bone regeneration

**FIGURE 7:**
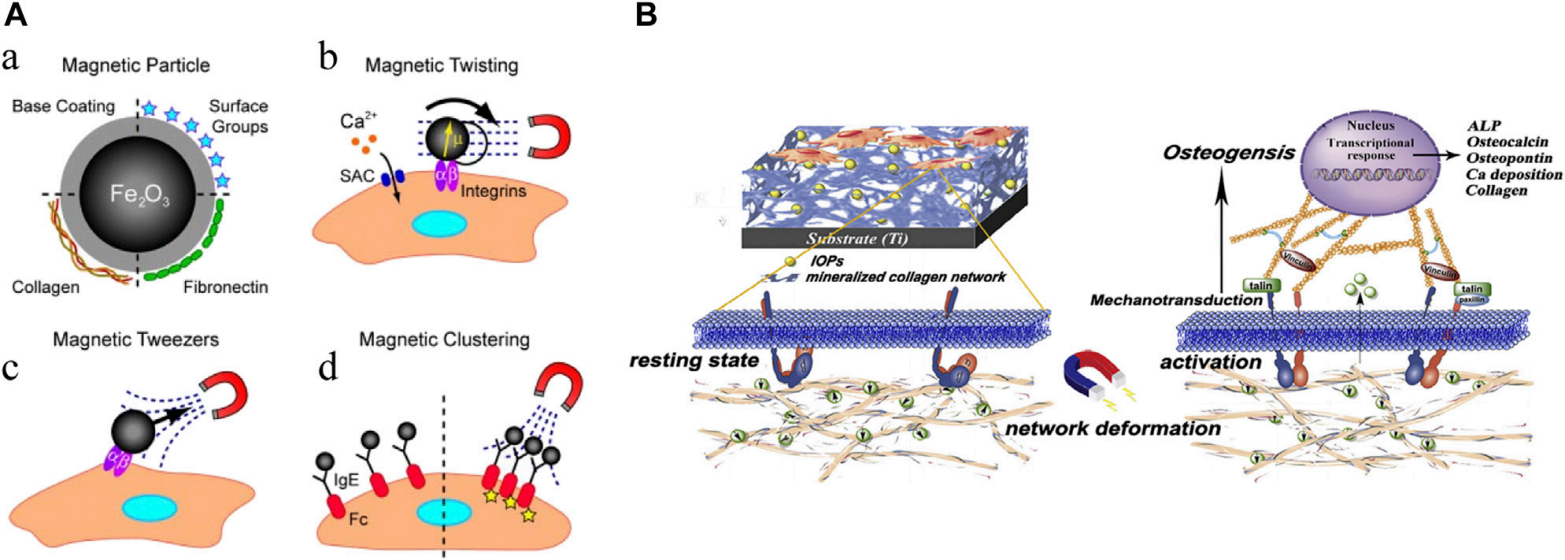
Mechanical stimulation of IONPs. **(A)** Mechanical activation of IONPs stimulates cellular receptors (Sniadecki, 2010); **(B)** Fe_3_O_4_/mineralized collagen coating promotes osteogenic differentiation of MC3T3-E1 cells (Zhuang et al., 2018).

**FIGURE 8:**
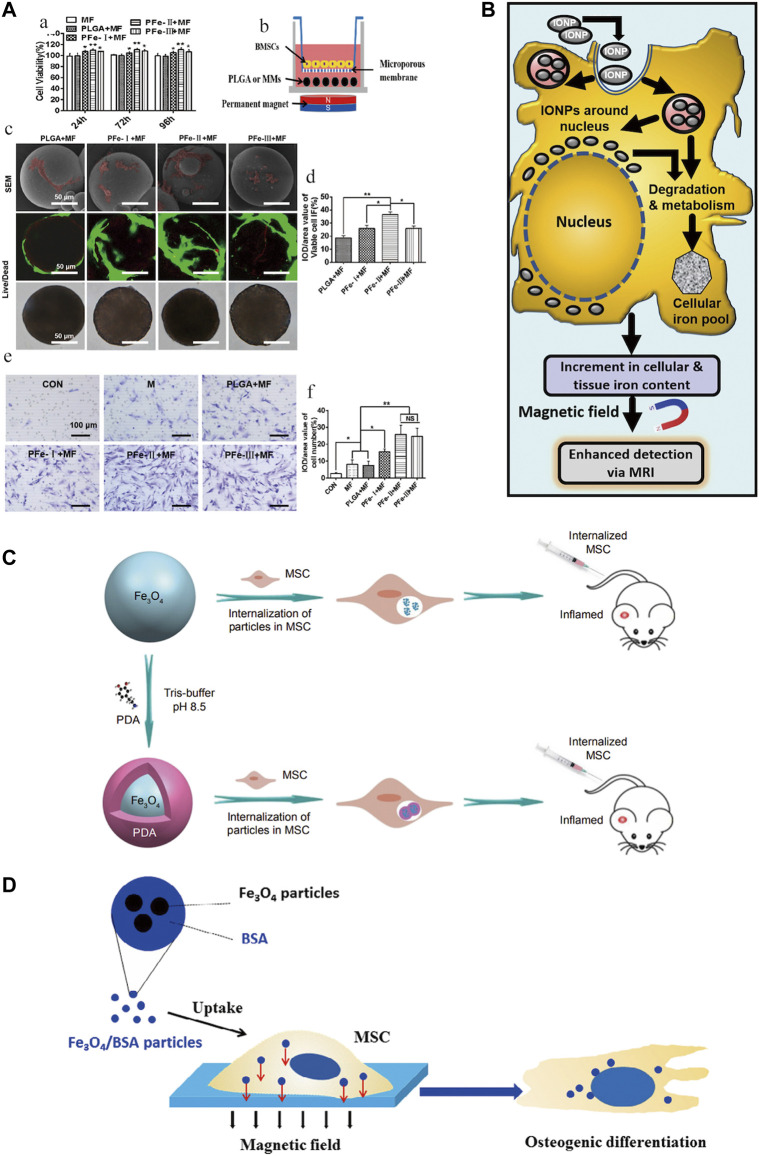
Role of IONPs on stem cells. **(A)** Promotion of stem cell migration (Zhao et al., 2021); **(B)** stem cell labeling (Mehta, 2022); **(C)** promotion of stem cell homing (Li et al., 2019); **(D)** promotion of stem cell differentiation (Jiang et al., 2016).

The multifaceted properties of IONPs have led to a wide range of research and applications in bone regeneration. The main directions of application include direct action (Figure 7), action on stem cells (differentiation, migration, homing, tracking) (Figure 8), targeted drug delivery, combined use with scaffolds, and composite scaffolds.

### Direct action

IONPs have a direct effect on cells. Various physical forces can affect cell activation, and studies have demonstrated that magnetic and mechanical stimulation can promote SCs proliferation and differentiation (Sniadecki, 2010; Schwartz et al., 2018). Magnetic forces can be converted into mechanical forces, and when cells are mechanically stimulated, they can generate a series of biochemical signals that induce a biological response to the organism (Ingber, 2006; Martino et al., 2018). Research has revealed that a composite coating formed by IONPs with a mineralized collagen coating under a static magnetic field can promote osteogenic differentiation through mechanical stimulation (Zhuang et al., 2018). Studies have also indicated that SIONPs themselves can strengthen bone regeneration without external magnetic fields (Hu et al., 2018). Proven research has also been conducted on the effects of the state of nanoparticles on cells, finding that direct delivery of colloidal ferromagnetic fluids is more desirable than the administration of powdered particles (Uskoković et al., 2019). Evidently, IONPs can stimulate cells directly with or without external magnetic field intervention and promote osteogenesis.

### Action on stem cells

SCs are an important tool in the treatment of bone defects. It holds great promise in treating many diseases and conditions (Daneshmandi et al., 2017; Česen Mazič et al., 2018). SCs are a particularly important group of cells in bone tissue engineering, which can differentiate into different cells under certain conditions. SCs are primarily derived from bone marrow, adipose tissue, muscle, umbilical cord, cord blood, placenta, Wharton’s glue, and amniotic fluid, all of which can differentiate into osteoblasts, of which the most studied are bone marrow mesenchymal stem cells (BMSCs) (Kangari et al., 2020). When bone damage occurs, MSCs migrate from the bone marrow or other adjacent tissues to the site of injury and secrete growth factors such as BMP and VEGF to promote the bone healing process (Schreivogel et al., 2019; Wasnik et al., 2019; McNeill et al., 2020). At the same time, MSCs are also involved in regulating inflammation and repairing bone damage together with progenitor cells, stromal cells and macrophages (Grayson et al., 2015; Xue et al., 2022; Yin et al., 2023). MSCs are also capable of differentiating into bone tissue and have favourable immunomodulatory properties (Safarova et al., 2020).

In recent years, SCs therapy has provided a strategy for bone regeneration, particularly in repairing large bone defects (Watanabe et al., 2016). As Hao et al. (2022) constructed microgels that can effectively promote the adhesion, proliferation, and osteogenesis of BMSCs, providing a new idea for repairing large bone defects. Liu et al. (Liu et al., 2022) also designed functional spheroids containing MSCs to create a 3D cell-directed microenvironment for the bone repair of large defects, resulting in excellent osteogenesis, angiogenesis, and bone regeneration of MSCs. For cells expanded *in vitro*, a stable cell phenotype should be maintained, targeting SCs to specific sites, promoting differentiation and proliferation of SCs to osteoblasts at the site of injury, maintaining local cell stability, and reducing cell loss at the defect site, all of which are necessary for bone defect repair.

### Stem cells differentiation

The differentiation of SCs is influenced by many factors, of which magnetic conditions are an important one. Ruchita et al. (Shelat et al., 2020) used L-lysine-functionalized IONPs to interfere with bone marrow mesenchymal stem cells (BMSCs) and showed efficacy in promoting *in situ* cartilage regeneration and its ability to label cells and act as a contrast agent. Driven by a magnetic field, carbon quantum dot (CD)-doped SPION can also differentiate MSCs into bone and cartilage for osteochondral regeneration (Das et al., 2019). Uptake of bovine serum albumin (BSA) (Fe_3_O_4_/BSA) particles loaded with IONPs also significantly enhanced osteogenic differentiation of MSCs under a constant static magnetic field (Jiang et al., 2016). Low-temperature atmospheric nitrogen plasma positively affects the behaviour of MSCs cultured on bone scaffolds containing IO-loaded silica nanoparticle catalysts, improving SCs behavior and promoting the value-added and osteogenic differentiation (Przekora et al., 2020). IONPs can directly promote the differentiation of SCs. However, current research tends to package and combine IONPs to strengthen this impact.

### Stem cells migration and homing

SCs labeled by IOPNs can migrate and home to specific sites in response to magnetic fields. It has been disclosed that the presence of SPIONs confers special magnetic properties to PLGA microspheres, which greatly promote the proliferation, migration, and osteogenic differentiation of BMSCs under the influence of external magnetic fields (Zhao et al., 2021). Moreover, the combination of pulsed electromagnetic fields (PEMF) and SPIONs were synergistic role in promoting the directional migration and osteogenic differentiation of BMSCs (Wu et al., 2018). A further finding was that silica-encapsulated IONPs promoted migration while retaining the proliferation and differentiation capacity of BMSCs and that the labeled BMSCs were more viable and migrated better, but their osteogenic potential was not affected (Yao et al., 2020). *In vitro* magnetic targeting system was used to attract rabbit BMSCs. This technique significantly facilitated the penetration of IONPs-labelled cells into porous hydroxyapatite ceramics transplanted into rabbit ulnar bone defects and promoted bone formation (Chen et al., 2010). IONPs enhance the homing potential of MSCs to the site of injury and have no negative impact on MSCs function; furthermore, MSCs-loaded nanoparticles exhibit good homing and anti-inflammatory capacity in the absence of external magnetic field intervention (Li et al., 2019). The combination of IONPs with MSCs has special magnetic effects and pro-osteogenic differentiation potential, which indicates the great ability of the two to merge for bone regeneration.

### Stem cell tracking

IONPs can be used as nuclear magnetic resonance (NMR) contrast agents for cell tracking (Hua et al., 2015). Currently, the commonly used MRI contrast agent is gadolinium, but its toxicity has raised the level of concern. When searching for an effective alternative without significant toxic effects, IONPs-labelled MSCs were found to show positive results both *in vitro* and *in vivo* (Mehta, 2022). Similarly, in a sheep tendinitis model treated with MSCs labeled with IONPs, cells were still detectable in MRI 7 days after surgery (Scharf et al., 2015). The magnetic effect mediated by IONPs is a crucial tool for SCs tracking and a wide range of applications. It has great potential as a magnetic particle imaging (MPI) tracer advancing SCs therapy (Wang et al., 2020).

### Targeted drug delivery

**FIGURE 9:**
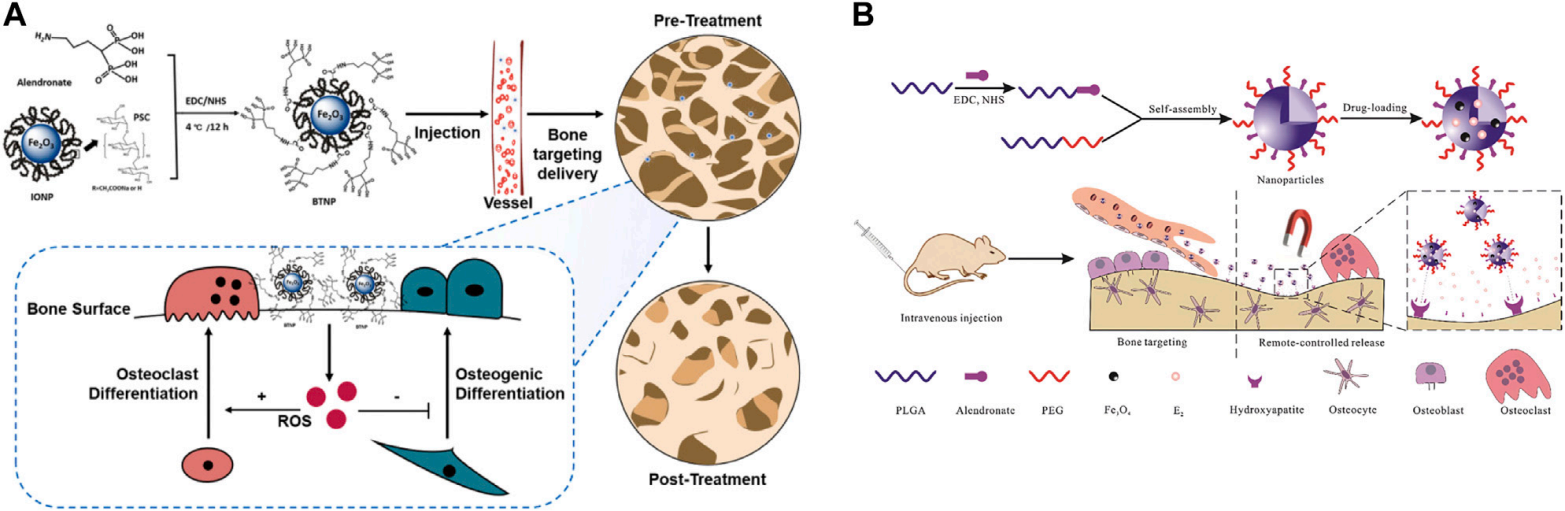
IONPs can be used for drug delivery. **(A)** Delivery of alendronate to osteoporosis (Zheng et al., 2022); **(B)** Remote controlled bone-targeted delivery of estradiol to treat ovariectomy-induced osteoporosis in rats (Guo et al., 2021).

Targeted drug delivery is an important feature of IONPs (Figure 9), and IONPs complexes are commonly used as carriers for controlled drug release (Prodan et al., 2013). For instance, IONPs have been found to stimulate the corresponding drug delivery systems for targeted drug delivery in arthritis (Zhang et al., 2022). To enhance the anti-osteoporosis effect, the investigators synergized IONPs with alendronate, and the combination substantially improved bone mineral density and microarchitecture compared to the same dose of alendronate (Zheng et al., 2022). Merging bisphosphonate (Bis) and IONPs and incorporating them into osteoblasts, this composite magnetic nanoparticle has the significant anti-osteoporotic potential (Lee et al., 2016). Guo et al. (2021) constructed a PLGA-based drug delivery system, co-loaded with 17β-estradiol (E2) and iron oxide (Fe_3_O_4_) and modified with alendronate for the remote targeting of estradiol in the treatment of post-ovariectomy osteoporosis in rats, which achieved good efficacy and magnetically remote targeted drug release. Besides, IONPs can be loaded with various cytokines, genes, and targets to reach the site of bone defects for the purpose of targeting and promoting bone regeneration. Jiang et al. (Chiang et al., 2018) developed magnetic gelatin nanocapsules containing transforming growth factor (TGF)-β1 consisting of hexanoic anhydride grafted gelatin and IONPs and achieved magnetic enrichment of loaded cells through combined treatment with magnetically induced stimulation and TGF-β1. Fan et al. (2014) developed a magnetic IONPs-encapsulated biopolymer nanogel consisting of chitosan and heparin via specific nucleobase pairing for Bone morphogenetic protein 2 (BMP-2) delivery, which plays an important role in cartilage and bone regeneration. For this purpose, Sahmani et al. (2020) generated a specific biomimetic scaffold with drug delivery capacity, a porous scaffold for biomedical use, prepared from hydroxyapatite and IONPs and loaded with gelatin, in which ibuprofen was incorporated to achieve excellent pain relief at the specified site. Researchers (Wang et al., 2021) have developed IONPs containing HA and raloxifene (R-IONPs-HA), which exhibit superior biocompatibility, antibacterial activity, and osteoinduction, and have great potential for fracture repair and infection prevention. Specific composite platforms are also being investigated for targeted drug delivery (Pistone et al., 2014). It is evident that the targeted delivery of IONPs is being refined and matured, and multiple compound platforms loaded with drugs have been investigated, showing a good ability to promote osteochondral regeneration.

### The role of the scaffolds

In bone tissue engineering exploration, scaffolds are the most used modality because they can retain specific structural and mechanical properties while loading other factors, particles, and materials, as well as being biocompatible and offering outstanding advantages in maintaining the normal physiological structure of bone and maintaining good weight-bearing and force-bearing functions. The design of delivery platforms plays a crucial role in the activation of bone regeneration as they provide a suitable environment for cell adhesion and growth while providing a beneficial platform for delivery strategies (Bi et al., 2020). Simple biodegradable polymeric materials or ceramics have been investigated as bone tissue engineering scaffolds, but these materials have significant limitations, including insufficient mechanical strength. In contrast, nanoparticle-modified composite scaffolds offer significant promise for promoting bone regeneration. The nanomaterials incorporated into polymeric scaffolds are divided into organic and materials, and they can provide sufficient surface area and mechanical properties required for support, as well as cell adhesion, differentiation, and integration (Kim and Fisher, 2007). The scaffolds currently in use mainly consist of organic materials (lipids, liposomes, dendrimers and polymers, chitosan, gelatin, collagen) or inorganic nano-biomaterials (silica, bioceramics, bioglass, hydroxyapatite) fused (Navarro et al., 2008; Virlan et al., 2016; Kumar et al., 2020). Xia et al. (2018a) mixes IONPs with calcium phosphate cement to create a scaffold that promotes osteoinduction and bone regeneration, adheres to SCs, and promotes osteogenic differentiation. In contrast, thermoplastic polyurethane (TPU) and PLA polymers doped with IONPs exhibited significant osteogenic differentiation of MSCs in response to external magnetic fields (Marycz et al., 2020). Hydrogels have excellent biocompatibility and adjustable mechanical properties, promising biomaterials for replacing extracellular matrix (ECM) and organ regeneration (Hu et al., 2015; Huang et al., 2017a; Xue et al., 2019). Han et al. (Han et al., 2021b) developed a tunable hydrogel for the 3D culture of dental pulp stem cells (DPSCs), through which DPSCs could aggregate and generate spheroids enriched with CD146^+^ cell subpopulations, which exhibited significant osteogenic differentiation in response to the intervention of IONPs.

**FIGURE 10:**
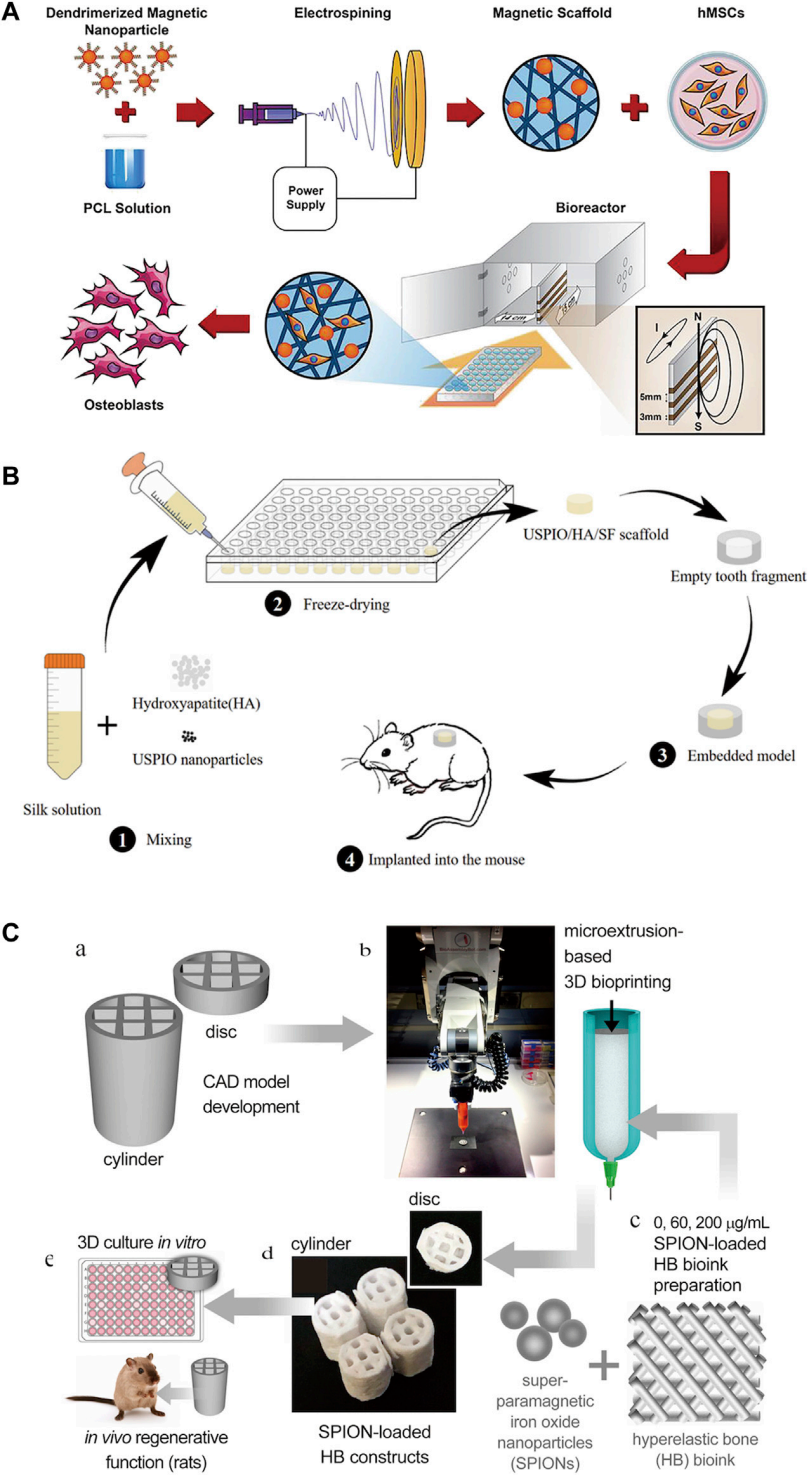
Three common methods for constructing scaffolds for IONPs. **(A)** Electrostatic spinning technique (Khalili et al., 2022); **(B)** freeze-drying technique (Zhang W. et al., 2019b); and **(C)** 3D printing technique (Shokouhimehr et al., 2021).

The mainstream methods currently used to synthesize scaffolds containing IONPs are electrospinning techniques, freeze drying and three-dimensional (3D) printing (Figure 10).

Electrospinning technology is a well-established scaffold fabrication method with many critical advantages, including its simple nature, high surface area to volume ratio, potential to release drugs and antimicrobial agents, controlled fiber diameter, high porosity, and permeability (Mortimer and Wright, 2017). To promote the fruitful differentiation of MSCs, a researcher prepared magnetic polycaprolactone (PCL) nanofiber scaffolds by adding third-generation dendrimer-modified SPIONs (G3-SPIONs) during electrospinning, which demonstrated promising results with favorable cytocompatibility and cell adhesion (Khalili et al., 2022). Although electrospinning technology is simple to handle and easy to control the surface properties of the support, it still has the disadvantage of low yield and low strength (Singh et al., 2014; Li et al., 2018b).

The freeze-drying technique contains both freezing and drying steps and is one of the most commonly used methods to enhance the stability of scaffolds (Rahman et al., 2010; Hassanajili et al., 2019). It is currently used as a common method for producing scaffolds in bone regeneration research. Freeze-dried scaffolds have a unique structure with the required porosity and pore interconnections for the selective movement of small molecules and closely resemble the natural bone extracellular matrix, making them optimal for bone tissue engineering studies (Singh and Dasgupta, 2022). Lu et al. (Lu et al., 2020) successfully prepared gadolinium-hyaluronic acid (Gd-HA) nanoparticles (NPs) by a simple freeze-drying method as a novel MRI contrast agent for the accurate detection of cartilage damage without toxic side effects. Wu Zhang et al. (2019b) used a combination of freeze-drying monthly physical mixing methods to prepare ultra-small superparamagnetic iron oxide (USPOI)-labeled hydroxyapatite (HA)/silk fibroin (SF) scaffolds loaded with dental pulp stem cells (DPSCs), which were physically stable and significantly promoted pulp tissue repair and regeneration. Azam et al. (Hajinasab et al., 2018) also applied freeze-drying methods to fabricate a 3D nanocomposite scaffold consisting of gelatin and hydroxyapatite (GEL/HA) loaded with Fe_3_O_4_ nanoparticles, which has great potential for drug loading and bone replacement. The flexibility of the freeze-drying technique can be seen to provide important support for the study of bone regeneration scaffolds.

3D printing methods allow the precise, reproducible, and large-scale manufacture of complex scaffold systems with tunable structural and physicomechanical properties (Filardo et al., 2019; Serpooshan and Guvendiren, 2020). 3D printing technology for bone tissue engineering also allows for specific geometric design of defective bone areas (Trombetta et al., 2017; Tomov et al., 2019). The manipulation of computer-aided design (CAD) models for printing scaffolds permits precise adjustment of the porosity, size, and geometric design of the graft to closely correspond to the target bone defect (Moreno Madrid et al., 2019). 3D printing technology offers good stability, 3D spatial structure, and high efficiency, but materials are more restricted and expensive (Zhao et al., 2014). Shokouhimehr et al. (2021) 3D printed a new generation of hyperelastic bone (HB) implants loaded with SPIONs applying a bio-ink consisting primarily of HA to scaffolds and studied their therapeutic effect on large non-healing fractures, demonstrating great potential for bone regeneration. The three scaffold fabrication methods and components are shown in Table 2.

**TABLE 2:** Fabrication type and research of scaffolds.

Manufacturing method	Coating	Magnetic field type	Cell type	Synthetic method	Nanoparticle type	Magnetic field intensity (emu/g)	Scaffolds proportion	References
Electrospinning techniques	PCL	PEMF	ADMSCs	Coprecipitation	G3-SPIONs	57.75	495 ± 144 nm	Khalili et al. (2022)
Electrospinning techniques	PCL	EMF	MC3T3-E1 cells	Coprecipitation	SPIONs	71	No application	Singh et al. (2014)
Freeze-drying technique	HA/SF	No application	DPSCs	Feeeze-drying	USPIO	No application	0.025 mg/ml USPIO and 5 mg/ml HA/SF	Wu Zhang et al. (2019b)
Freeze-drying technique	GEL-HA	EMF	L929 cells	Feeeze-drying	Fe_3_O_4_ nanoparticles	49.6	No application	Hajinasab et al. (2018)
3D printing methods	HA	No application	C3H10T12 cells and HBO cells	3D printing	SPIONs	No application	SPION concentration between 60 and 200 µ g/ml	Shokouhimehr et al. (2021)

### The role of the composite scaffolds

**FIGURE 11:**
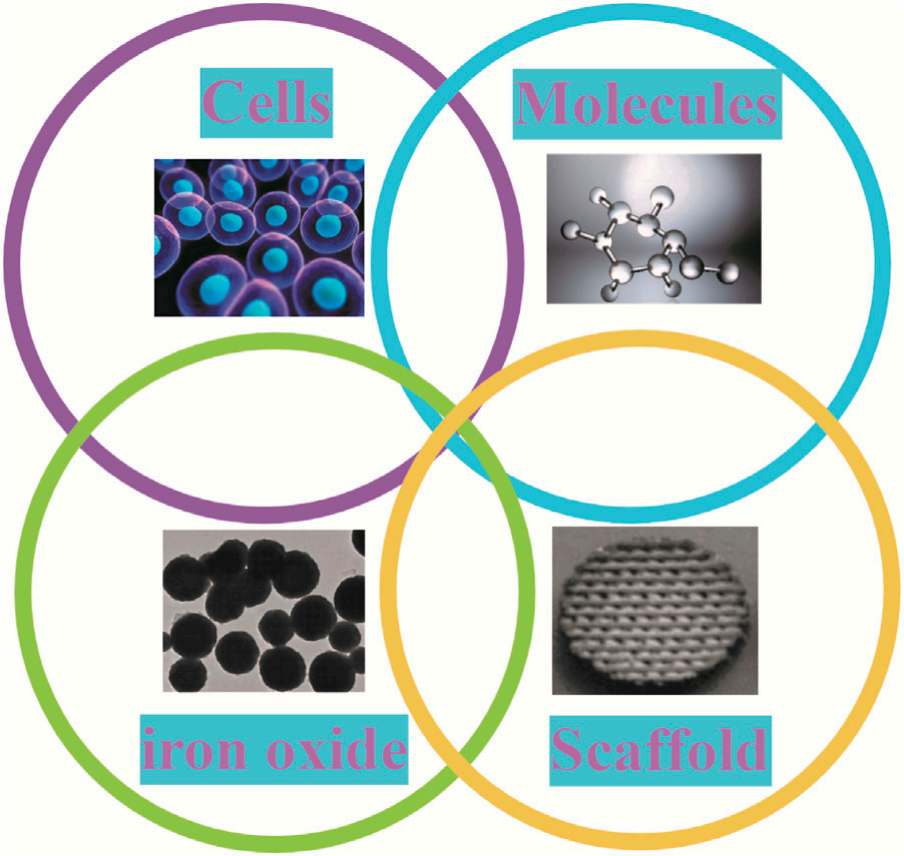
Components of tissue engineering in IONPs. When IONPs are applied to tissue engineering, this body has four main components: cells, molecules, IO and scaffold.

**FIGURE 12:**
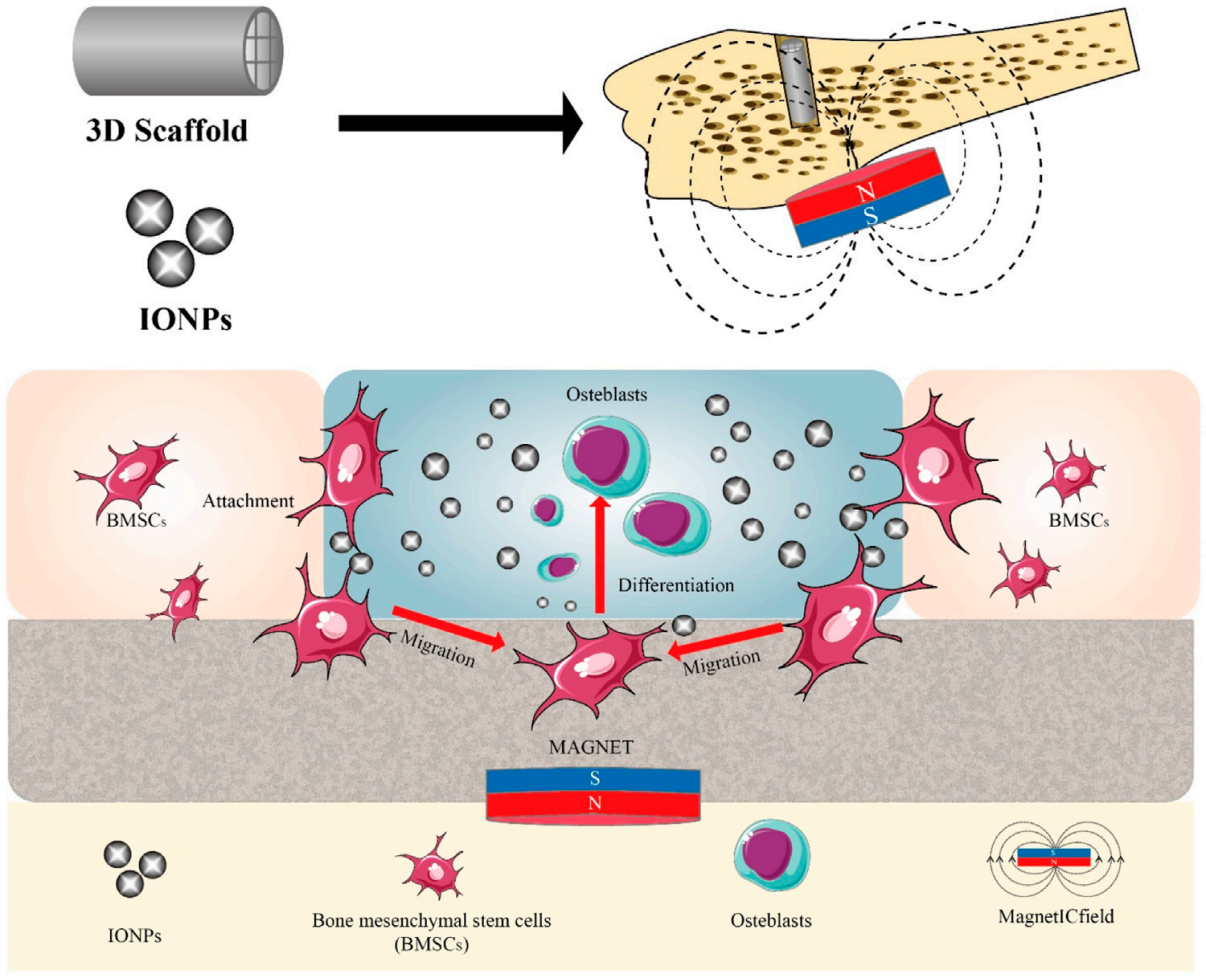
Application of composite scaffold in bone regeneration. IONPs are mostly found in the form of scaffolds in the treatment of bone defects. To enhance their efficacy, scaffolds can contain a variety of components that promote osteogenic differentiation of stem cells under the influence of a magnetic field.

Tissue engineering is a method of reconstructing functional tissue at the site of damage, usually including cells, growth factors, and 3D biodegradable scaffolds. With the addition of IONPs to the tissue project, it will contain four main components (Figure 11). Its complex construction is of great importance in promoting bone regeneration. In order to strengthen the effectiveness of the bone repair, extend the synergistic effect and reduce the deficiencies possessed by a single scaffold, researchers are now primarily developing composite scaffolds to reach the desired goal (Figure 12). Researchers combined aminopropyltriethoxysilane (APTES)-modified nanohydroxyapatite (nHAp) with IONPs to develop composites that significantly enhanced osteoblast activity and reduced inflammatory responses and osteoclastogenesis (Marycz et al., 2022). By coating core-shell structured HA on SPIONs (SPIO@HA), Li et al. (Li M. et al., 2021) discovered it could target osteoclastogenesis and osteogenesis. A multifunctional scaffold of filamentous protein/hydroxyapatite scaffold combined with ultra-small SPIONs (USPIO) doped with BMSCs promotes osteogenic differentiation of SCs and allows for osteogenic differentiation non-invasive monitoring of bone regeneration by quantitative MRI (Liu Q. et al., 2020). Moreover, oleic acid-modified IONPs (IO-OA NPs) and PLGA were used to produce homogeneous magnetic nanocomposites, which showed good mechanical properties and enhanced osteogenic differentiation (Hao et al., 2019). In addition, calcium phosphate cement (CPC) scaffolds doped with IONPs on SCs utilizing an external static magnetic field (SMF) have great osteoinductivity, which may be related to the physical forces generated by the magnetic field and the cell-intrinsic action of the magnetic nanoparticles released from the scaffold (Xia et al., 2019a). The relevant modification of the surface of IONPs and their subsequent application to composite scaffolds is also a way of extending their efficacy. Kartogenin (KGN), an emerging stable non-protein compound that promotes the differentiation of BMSCs into chondrocytes, was transplanted onto the surface of modified USPIO and then made into a fibrin nanocrystal/dextran hydrogel, which showed enhanced cartilage regeneration (Yang et al., 2019).

Bone tissue-engineered scaffolds alone have many drawbacks, including lack of osteoinduction and low mechanical strength. To circumvent these limitations, the option of composite scaffolds is necessary. Hu et al. (Hu et al., 2021) used a poly (lactic acid-ethanolic acid copolymer)/polycaprolactone/β-tricalcium phosphate (PPT) scaffold, incorporating IONPs into a porous electromimetic scaffold for long-term release, and this composite scaffold was optimized to further enhance osteogenic capacity. The low mechanical strength of the scaffold can be regulated by various preparation techniques, of which electrostatic spinning is one of the most applied (Schiffman and Schauer, 2008; Jun et al., 2018). Chen et al. (2018) reported magnetic PLGA/PCL scaffolds made by electrostatic spinning technology and layer-by-layer assembly of SPIONs, which exhibit excellent biocompatibility, adjustable stiffness, physical sensing, and stimulus responsiveness. Estévez et al. (2022) were the first to combine type I collagen and SPIONs to design magnetic and biocompatible electrostatic spinning scaffolds, showing that the electrostatic spinning structures containing SPIONs could retain the magnetic properties of the nanoparticles alone and that these collagen scaffolds could promote the proliferation, adhesion, and viability of osteoblasts and HBMSCs.

3D printing technology for composite scaffolds is another commonly used method whose versatility makes it increasingly important in bone regeneration. The study found that IONPs-rich PLGA copolymer scaffolds prepared through the layer-by-layer assembly by 3D printing technology promoted cell adhesion and enhanced osteogenesis of rat BMSCs (Han L. et al., 2021). Saraiva’s group (Saraiva et al., 2021) used 3D printing to create a polylactic acid platform loaded with HA, IONPs, and an antibiotic (minocycline) with tunable properties and a multi-stimulus response. The platform was discovered to regulate the anti-biofilm dose and strengthen the ability of bone tissue regeneration. Utilizing 3D printing technology, porous titanium aluminium vanadium (Ti6Al4V, pTi) scaffolds can reconstruct large bone defects (especially in load-bearing areas). Nevertheless, the lack of osteogenic properties limits its use in clinical practice. To address this dilemma, a researcher co-deposited IONPs and polydopamine (PDA) on the surface of 3D printed pTi scaffolds, a novel magnetic coating that improved the adhesion, proliferation, and osteogenic differentiation of HBMSCs (Huang et al., 2020). Wang et al. (2018) synthesized biodegradable shape memory polyurethane (PU) as the main component of a 3D printing ink for manufacturing bone scaffolds and added SPIONPs and polyethylene oxide (PEO) or gelatin to the 3D printing ink to promote osteogenic induction, shape fixation and increase printability. This composite scaffold with its shape memory properties, biodegradability, and osteogenic effect, has the potential to become a bone substitute. Petretta et al. (2021) prepared a polycaprolactone-based (PCL) scaffold with HA and SPIONs (PCL-HAp-1% SPION) by 3D printing to improve the healing efficiency of bone and inoculated the scaffold with MSCs.

The construction of composite scaffolds gives new ideas for bone regeneration, while the addition of drug loading after the platform has been established can achieve certain therapeutic effects for specific bone diseases. As Schneider et al. (Schneider et al., 2021) used citric acid (MG@CA) coated magnetic iron oxide nanoparticles (MNPs) as a raw material for the treatment of bone diseases to construct a magnetic nanoplatform, adding raloxifene (Ral), curcumin (Cur), and methylene blue (MB) for the symptomatic treatment of bone diseases.

## Application of iron oxide nanoparticles in pathological bone defects

Mild bone injuries have some ability to heal themselves, but beyond a certain point, bone defects usually fail to reconstruct themselves. For this type of bone defect that does not heal on its own, pathological bone defects such as osteoporosis (Bai et al., 2020; Li et al., 2022a; Li et al., 2022b), rheumatoid arthritis (Liu et al., 2021), infections (Taylor and Webster, 2011) and tumors (Kesse et al., 2020) are the main causes. In contrast, IONPs have been extensively investigated in pathological bone defects. Several studies have demonstrated that IONPs can positively regulate bone metabolism *in vitro* and *in vivo*, and that they have favorable bone targeting, bone regeneration and biocompatibility properties. For instance, Zheng et al. (2022) revealed that IONPs can alleviate osteoporosis induced by ovariectomized mice (OVX) by scavenging reactive oxygen species, promoting osteogenic differentiation of BMSCs and inhibiting osteoclastogenesis *in vitro*. Krzysztof et al. (Marycz et al., 2022) combined aminopropyltriethoxysilane (APTES) modified nHAp with IO nanoparticles and demonstrated *in vitro* that this biomaterial promotes osteoblast viability and RUNX-2 expression, reduces osteoclast metabolism and inflammation, and can be used for healing of osteoporotic fractures. Another study produced a SPIO with a core-shell structure coated with HA (SPIO@HA), which was used to target osteoclastogenesis and osteogenesis. The results revealed that the material greatly prevented bone loss in OVX mice and that their mechanism may be related to TRAF6-p62-CYLD, TGF-β, PI3K-AKT and calcium signalling pathways (Li et al., 2021a). ​Moreover, IONPs can be used for drug delivery in the presence of magnetic fields, bone targeting for osteoporosis (Guo et al., 2021).

Rheumatoid arthritis (RA) is a systemic immune disease that is primarily characterised by joint inflammation accompanied by progressive bone and joint destruction, which can cause significant bone defects (Takeuchi et al., 2021). Magnetic nanoparticles, particularly IONPs, have been documented to be an important tool for the treatment and diagnosis of rheumatoid arthritis by targeting local lesions with additional magnetic fields and avoiding systemic side effects (Liu et al., 2021). IONPs are an effective nanoplatform for targeted drug delivery, stimulus-responsive drug release, MRI diagnostics, photothermal therapy, and magnetothermal therapy in RA treatment, and could also contribute to the development of new magnetic materials (Wu and Shen, 2019). Duan et al., (2014) synthesized polyethyleneimine-functionalized superparamagnetic iron oxide nanoparticles (PEI-SPION) *in vitro* and demonstrated through *in vivo* and *in vitro* experiments that PEI-SPION can be used for systemic siRNA delivery to strengthen the therapeutic effect of RA with the intervention of an external magnetic field. Furthermore, Carneiro et al. (2020) constructed gold-coated SPIO (AuSPION) for the treatment of arthritic rats by intraperitoneal injection and found that AuSPION inhibited joint inflammation and edema, redox imbalance and cytokine expression under the influence of an external magnetic field.

Infection is another major challenge for bone regeneration, but this has been made viable with the intervention of nanotechnology. Studies have indicated that SPIONs have good antimicrobial activity, magnetic properties, and bone regeneration properties, which could be used in the development of new drugs for the treatment of bone defects associated with orthopaedic infections (Taylor and Webster, 2011). Wang et al. (2021) used IONPs (R-IONPs-HA) containing HA and raloxifene for fracture repair and infection prevention which showed good biocompatibility, antibacterial activity and osteoinductive effects. Taylor et al. (Taylor and Webster, 2009) demonstrated in an *in vitro* study that SPION significantly inhibited the growth of *Staphylococcus* epidermidis and at the same concentration significantly enhanced osteoblast function, offering hope for bone repair after infection. Wei et al. (2021) synthesized IONPs-sHA-strontium@collagen (IONSs-HA-SR@C) using a co-precipitation method and evaluated its effect on the differentiation and proliferation of MC3T3-E1 cells and their morphological changes as well as its antibacterial activity by *in vitro* experiments. The results clearly indicate that IONSs-HA-SR@C has promising biocompatibility, bone growth promoting ability and antibacterial activity. It is evident that IONPS has great potential for bone repair and anti-infection.

The incorporation of IONPs into multifunctional biomaterials for the treatment of tumors and post-tumour bone defects has led to a number of advances and breakthroughs due to their thermal, magnetic targeting, bone healing and pro-tumour cell apoptosis properties. Pang et al. (2021) synthesized a bone-targeted SPIO that, when delivered to bone, inhibits flavoproteinase, thereby inhibiting bone resorption by osteoclasts and invasion by breast cancer cells, effectively protecting against breast cancer-induced osteolysis. Khodaei et al. (2022) developed a method for the delivery of curcumin by SPIONs-based magnetothermal nanocarriers, in which the release of curcumin was coordinated by temperature. *In vitro* results showed that the temperature could be stabilised at 41°C by alternating magnetic fields, and that curcumin and heat therapy could kill MG-63 osteosarcoma cells and achieve good anti-bone tumour effects. Not only that, IONPs can promote bone regeneration for the repair of bone defects after tumour surgery. Sasikala et al. (2022) developed a piezoelectric magnetic nanoparticle (PMNP) that can regulate cell behaviour and fate under ultrasound (US), magnetic field (MF) and light-responsive conditions to achieve superior bone regeneration and bone transformation after bone tumour surgery. Vergnaud et al. (2022) designed superparamagnetic bioactive nanoparticles with an IO core (γ-Fe2O3) wrapped in a bioactive glass (SiO2-CaO) shell, which demonstrated *in vitro* experiments its non-toxic, bone healing and thermal therapeutic properties for direct or combined scaffolding of bone defects after bone tumour resection. It is apparent that IONPs have a great platform and opportunity in the treatment of pathological bone defects and have now gained great traction.

## Mechanism of iron oxide nanoparticles in bone regeneration

**FIGURE 13:**
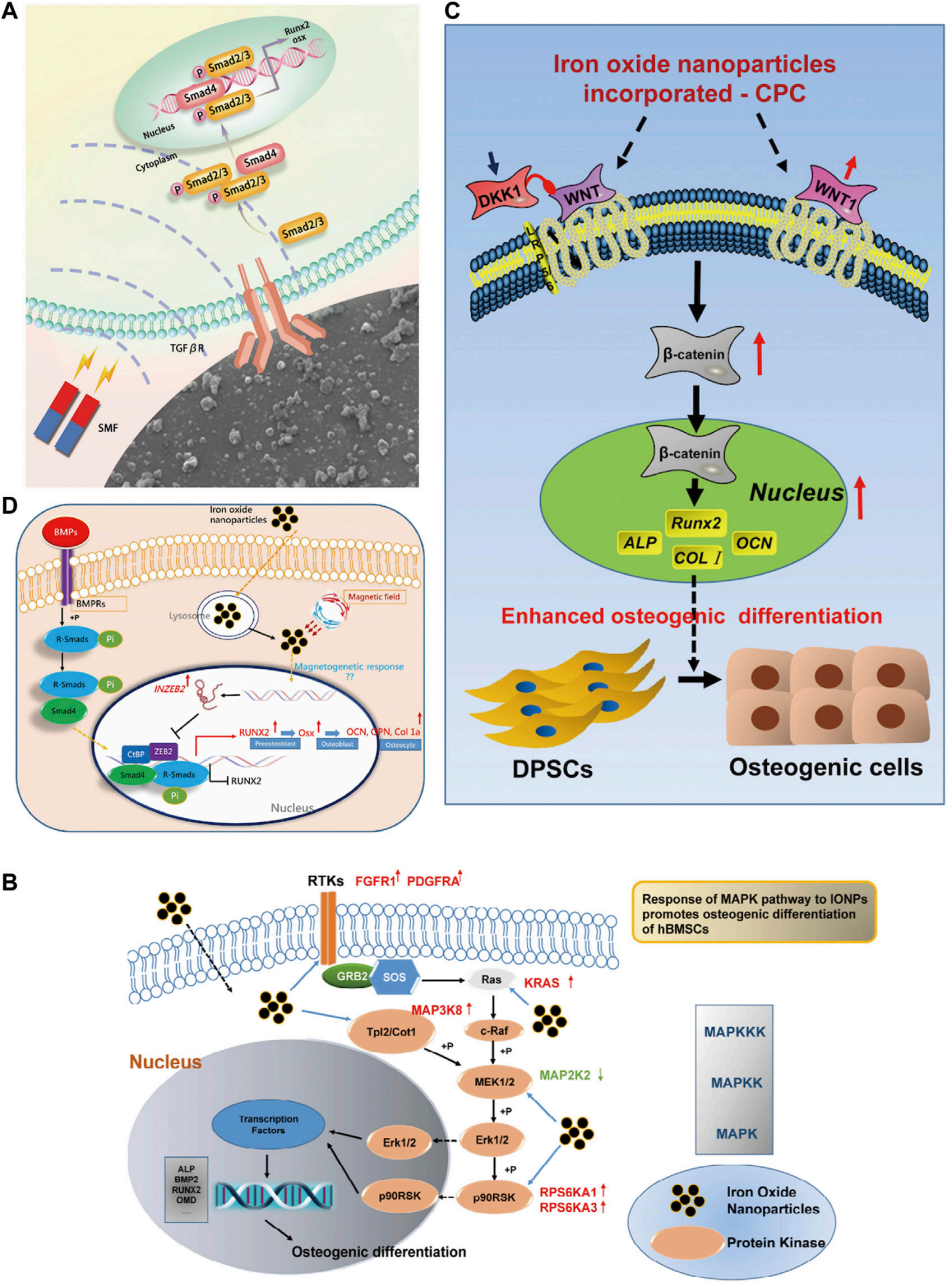
Mechanism of IONPs in the treatment of bone defects. **(A)** In the action of SMF, the Fe3O4/PDA@pTi scaffold regulates cellular function via the TGF*β*-Smads signaling pathway (Huang et al., 2020); **(B)** IONPs promote osteogenic differentiation of HBMSCs through the MAPK pathway when they enter the cells (Wang et al., 2016); **(C)** IONPs + CPC can promote osteogenic differentiation of HDPSCs through the WNT signalling pathway (Xia et al., 2019b); **(D)** IONPs promote osteogenic differentiation of MSCs through the INZEB2 and BMP/Smads signaling pathways (Wang et al., 2017).

The rational use of IONPs can better promote bone regeneration. Previous studies have focused on the construction of scaffolds, the selection of composite materials, and the integrated use of SCs and drugs but neglected their specific mechanisms. In recent years, with the development of molecular biology, the study of the mechanism has gradually applied to the field of materials; the results found that IONPs promote bone regeneration with a variety of factors and signaling pathways related (Figure 13). Proteomic analysis revealed that magnetic iron oxide/polydopamine coating (Fe_3_O_4_/PDA) promotes osteogenesis by upregulating the TGFβ-Smads pathway in a static magnetic field (Huang et al., 2020). Heat therapy is beneficial in stimulating bone regeneration. Wang et al. (2022) constructed nanoparticle-hydrogel composites by embedding Arg-Gly-Asp (RGD)-coated core-shell structured magnetic iron oxide nanoparticles (MION) (CoFe2O4@MnFe2O4) in agarose with significant magnetothermal effects. Under mild magnetothermal treatment, the expression of HSP90 was upregulated in preosteoblasts and endothelial cells, activating the PI3K/Akt pathway in preosteoblasts, thus significantly promoting osteogenesis and biomineralization; meanwhile, the cobalt in CoFe2O4@MnFe2O4 upregulated the expression of the angiogenesis-related gene HIF-1 α, further promoting the formation of new blood vessels at the injury site. Zhuang et al. (2018) incorporated IONPs into mineralized collagen coatings (MC) to promote osteogenic differentiation by initiating mechanotransduction pathways and upregulating integrin α1, integrin β1 and RhoA under static magnetic fields. Jia et al. (2019) synthesized mesoporous silica-coated magnetic (Fe_3_O_4_) nanoparticles (M-MSN), which exhibited excellent biocompatibility and promoted osteogenic differentiation of MSCs *in vitro* via the Wnt/β-catenin pathway. In healing osteoporosis, IONPs can inhibit osteoclastogenesis by modulating the TRAF6-p62-CYLD signaling complex (Liu et al., 2020a). Yu et al. (2020) synthesized Fe_2_O_3_@PSC nanoparticles using the biopolysaccharide-based antioxidant polyglucose-sorbitol-carboxymethyl ether (PSC) as a precursor and found that it scavenged reactive oxygen species from MC3T3-E1 and Raw 264. cells by activating the Akt-GSK-3β-β-catenin pathway, inhibiting MAPK and NF-κB pathways ultimately promoting osteoblast differentiation. IONPs, in combination with SCs or biomaterials, will exhibit multiple functions. It was discovered to promote osteogenic differentiation of hBMSCs via the MAPK pathway (Wang et al., 2016). Jiang et al. (2016) prepared bovine serum albumin (BSA) particles loaded with IONPs (Fe_3_O_4_/BSA) and found that they significantly promoted the expression of alkaline phosphatase, type I collagen, and osteocalcin at the mRNA and protein levels in MSCs under static magnetic fields. The microbiota influences the process of bone repair. By constructing a rat palatal bone model, Jia et al. (2021) found that 3D printed SPION/PLGA could have a significant antibacterial effect and could alter the oral microbiota while treating palatal bone defects. Lu et al. (2018) prepared magnetic SrFe_12_O_19_ nanoparticle-modified mesoporous bioglass (BG)/chitosan (CS) porous scaffolds (MBCS) with excellent bone regeneration and anti-tumor functions. The magnetic field generated by MBCS can promote the expression of osteogenic genes such as OCN, COL1, Runx2, and ALP by activating the BMP-2/Smad/Runx2 pathway; it can also perform photothermal conversion to promote apoptosis of tumour cells, so it is used for tumor-associated bone defects. The combination of IONPs, MSCs, and biomaterials to build a composite system is the mainstay of bone regeneration research, and some breakthroughs have been made in their mechanisms. Duan et al. (2020) constructed a human umbilical cord mesenchymal stem cell-super magnetic iron oxide nanoparticles (NPs)@polydopamine (SCIOPs) system, which was found to inhibit osteoblast apoptosis, enhance osteoblast proliferation and promote bone repair through the Akt/Bcl-2/Bad/caspase-3 signaling pathway in the presence of a magnetic field and SCs homing ability. Xia et al. (2019b) added IONPs powder to CPC powder to fabricate CPC-IONP scaffolds and investigated the effect of the novel composite on bone matrix formation and osteogenesis of human dental pulp stem cells (hDPSCs). The results indicate that the osteogenic behavior of hDPSCs may be driven by CPC-IONP via the WNT signaling pathway. The mechanism by which IONPs promote bone repair has also been explored from the perspective of RNA. It was found to upregulate lncRNA INZEB2, regulating ZB2 expression and the BMP/Smads pathway (Wang et al., 2017). As nanomaterials are increasingly used in bone regeneration, it is essential to understand their biological effects (Table 3). The ongoing exploration of their molecular mechanisms may provide a theoretical basis and biological evidence for regenerative medicine.

**TABLE 3:** Key elements of the study on the mechanism of IONPs for bone defects.

Compound component	Experimental method	Animal model	Cell type	Magnetic properties	Signaling pathway	References
Fe_3_O_4_/PDA	*In vivo* and *in vitro*	Rabbit model of femoral epicondyle defect	hBMSCs	SMF	TGFβ-Smads signaling pathway	Huang et al. (2020)
IONPs(Feraheme)	*In vivo* and *in vitro*	Ovariectomized (OVX) mice	RAW264.7 macrophages, bone marrow monocytes (BMMS), osteoclasts	No application	Inhibit RANK signaling pathway by regulating TRAF6-p62-CYLD signaling complex	Li Liu et al. (2020)
IONPs-PSC	*In vitro*	No application	hBMSCs	No application	MAPK signaling pathway	Wang et al. (2016)
Fe_3_O_4_/BSA	*In vitro*	No application	rBMSCs	SMF(1T)	Alkaline phosphatase (ALP) activity, calcium deposition, type I collagen and osteocalcin	Jiang et al. (2016)
SPION@PDA NPs (Fe_3_O_4_)	*In vivo* and *in vitro*	Rats were injected with 20 mg/kg/d methylprednisolone in the muscle above the femoral head for 3 days (rat femoral head necrosis model)	HU-MSCs	SMF	Akt/Bcl-2/Bad/caspase-3 signaling pathway	Duan et al. (2020)
IONP-CPC (Fe_2_O_3_)	*In vitro*	No application	hDPSCs	No application	Wnt/β-catenin signaling pathway	Xia et al. (2019b)
IONPs-PSC	*In vitro*	No application	hBMSCs	No application	BMP/Smad signaling pathway	Wang et al. (2017)
IONP-Exos (Fe_3_O_4_)	*In vivo* and *in vitro*	Anterior cruciate ligament resection model in rats	hBMSCs, NIH3T3 fibroblasts	SMF	miR-21-5p/SMAD7 signaling pathway	Xiang-Dong Wu et al. (2022)
M-MSNs (Fe_3_O_4_)	*In vivo* and *in vitro*	Rat tibia DO model	rBMSCs	No application	Wnt/β-catenin signaling pathway	Jia et al. (2019)
IOP-MC	*In vitro*	No application	MC3T3-E1 cells, rBMSCs	SMF	Mechanotransduction signaling pathway (integrin α1, integrin β1 and RhoA)	Zhuang et al. (2018)
MBCS (SrFe_12_O_19_)	*In vivo* and *in vitro*	Bilateral critical-sized calvarial-defect models in rat	hBMSCs, MG-63 cells	SMF	BMP-2/Smad/Runx2 signaling pathway	Lu et al. (2018)

## Hot spots and trends in iron oxide nanoparticles for bone regeneration

**FIGURE 14:**
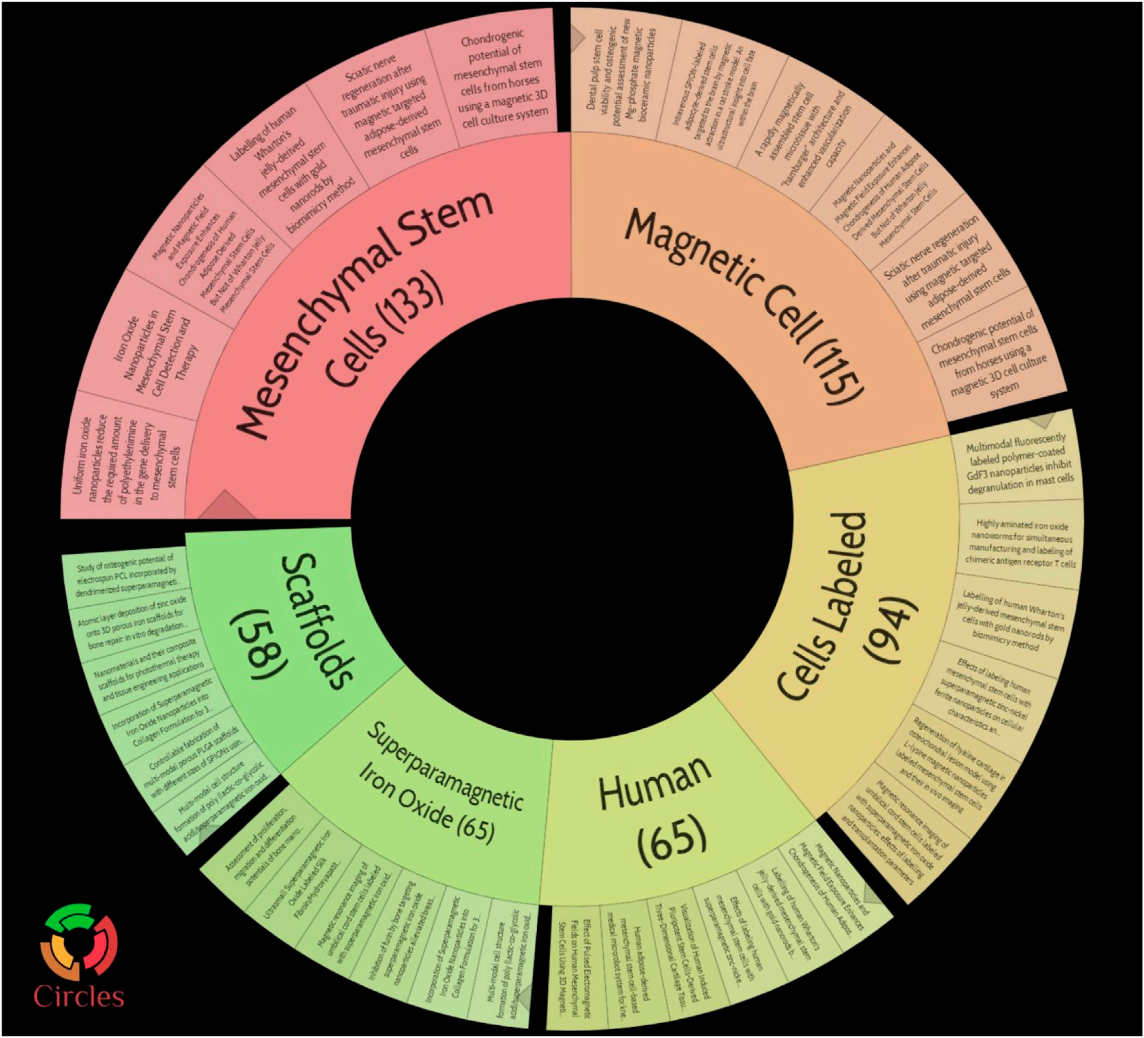
Pie chart of topic clustering in carrot2. The main directions of research clustering are Mesenchymal Stem Cells, Magnetic Cells, Cells Labeled, Human, Superparamagnetic Iron Oxide, and Scaffolds.

**FIGURE 15:**
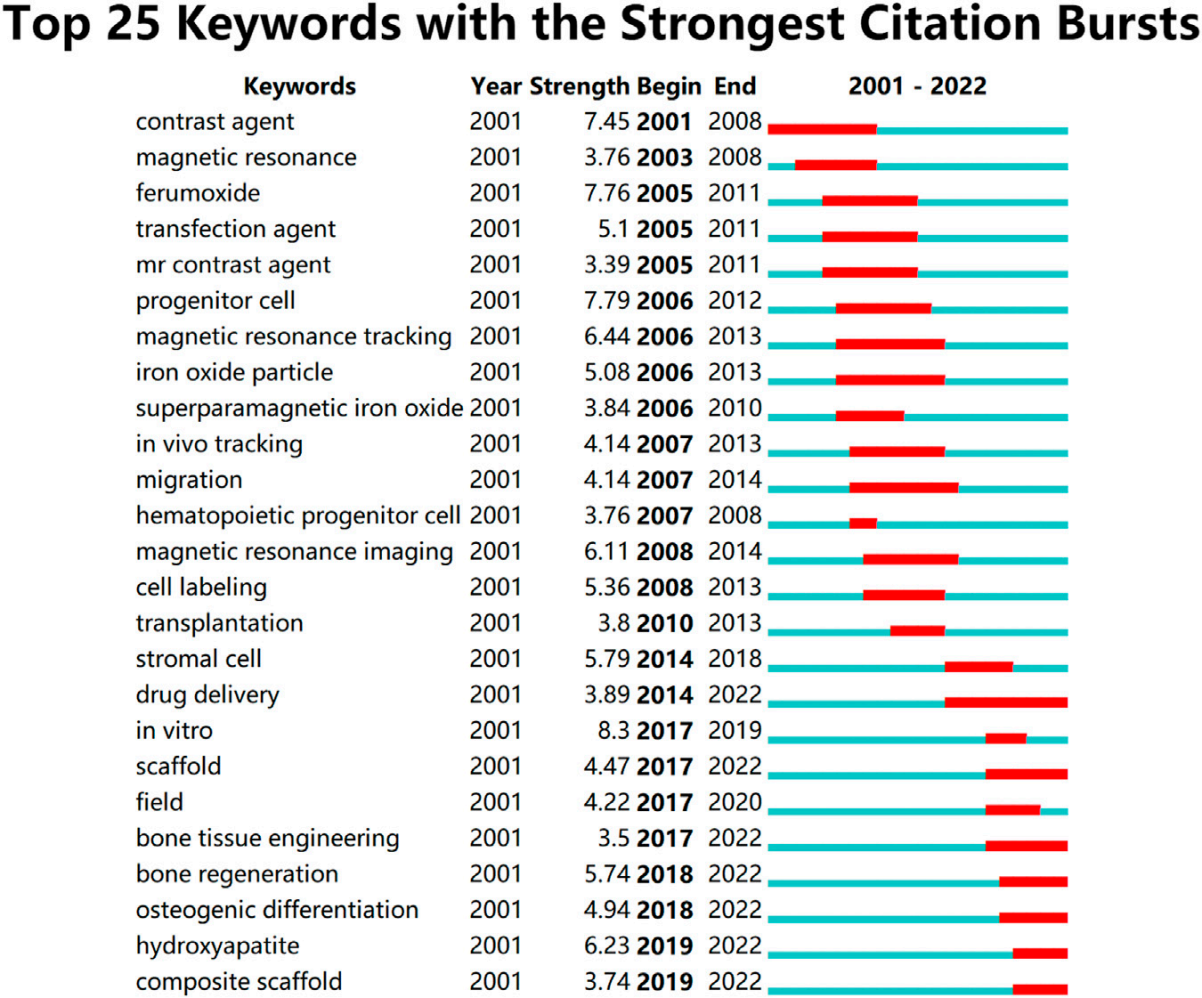
Top 25 keywords with the strongest citation bursts. This research focuses on three main blocks of research: stem cell research, cell marker research, and research on scaffolds. Based on the analysis of the strongest citation bursts of keywords, the development of this study can be divided into three main periods. In the early stages, research in this field concentrated on MRI and diagnostic aspects of disease; in the middle stages, it focused on the labeling of SCs and *in vivo* tracking; the current hotspots and trends are in scaffold research, regenerative tissue engineering of bone and osteogenesis, and the transformation of the scaffold from a single component at the beginning to a composite scaffold.

A bibliometric approach was employed to investigate the hotspots and trends in IONPs on bone regeneration. We searched the Web of science core database for 631 relevant primary literature and, after limiting the language of the literature to English, eventually obtained 629 publications for inclusion in the study. The literature was imported into the Carrot2 clustering analysis tool to identify the current mainstream research directions. A burst analysis of the involved literature was also carried out using Citespace software to identify hot spots and trends in recent years. The results obtained from the Carrot2 analysis revealed that the main directions of research clustering are Mesenchymal Stem Cells, Magnetic Cells, Cells Labeled, Human, Superparamagnetic Iron Oxide, and Scaffolds (Figure 14). Burst analysis disclosed that IONPs were initially applied as magnetic resonance imaging contrast agents and transfection agents and then steadily moved towards magnetic resonance and *in vivo* tracking. After incorporating stem cells into the field, IONPs tend to be focused on migration, cell labeling, transplantation, and drug delivery. The increasing use of biomaterials has led to the development of IONPs from *in vivo* tracking to *in vitro* studies. Recent years have seen a shift towards the scaffold, bone tissue engineering, osteogenic differentiation, hydroxyapatite, and composite scaffold (Figure 15). The results of the thematic clustering reveal that the research literature in this area focuses on three main blocks of research: stem cell research, cell marker research, and research on scaffolds. Based on the analysis of the strongest citation bursts of keywords, the development of this study can be divided into three main periods. In the early stages, research in this field concentrated on MRI and diagnostic aspects of disease; in the middle stages, it focused on the labeling of SCs and *in vivo* tracking; the current hotspots and trends are in scaffold research, regenerative tissue engineering of bone and osteogenesis, and the transformation of the scaffold from a single component at the beginning to a composite scaffold. It is worth mentioning that HA is the main inorganic component of bone and its excellent biocompatibility, osteoconductivity and osteoinductivity, binding similarity, and high porosity make it an important area of interest for bone regeneration research (Ramesh et al., 2018; Gomes et al., 2019). Its effects are primarily in the coating of IO, the construction of scaffolds, and the delivery of drugs (Khajuria et al., 2018). However, HA has certain drawbacks, as its fragility reduces the mechanical properties of the material, and its demonstrated difficulty in modification makes the nanoparticles prone to agglomeration (Jegatheeswaran and Sundrarajan, 2015; Alaribe et al., 2016). To address these issues, nHAp can be combined with polymers to strengthen their mechanical properties, biocompatibility, and biofunctionability, eventually improving overall regeneration (Song et al., 2016).

## Concluding remarks—future perspectives

Although bone has the potential to heal itself, larger bone defects are irreversible; it is a serve health problem and how to promote bone regeneration remains a clinical challenge. IONPs are an important member of the nanoparticle class because their magnetic properties give them unique advantages in bone repair, including stimulating osteogenic differentiation, cell labeling, drug delivery, molecular activation, and SCs homing. The powerful effect of IONPs is inseparable from their coating. The external coating reduces the toxicity of IO and improves the overall biocompatibility and mechanical properties of the IONPs. Additionally, the external modification of the coating with cytokines, drugs, and targets can also give IONPs a targeted therapeutic effect. The bone regenerative effect of IONPs is also influenced by external magnetic fields, which can be remotely controlled to induce various biological responses. IONPs are now regularly used in combination with SCs and scaffolds to achieve good bone regeneration results. It promotes migration, adsorption, homing, labeling, and osteogenic differentiation of SCs and confers a magnetic effect on the scaffold. In composite scaffolds, good biocompatibility, mechanical properties, resorbability, osteoconductivity, and osteoinductivity are essential for bone regeneration. Although some scholars have initiated to explore the specific mechanisms by which IONPs treat bone defects, the interactions between magnetic fields, cells, and nanoparticles in composite scaffolds are still not sufficiently understood. Although basic research has identified the crucial role of bone morphogenetic proteins, vascular endothelial growth factor, osteocalcin, and collagen in bone regeneration, there is still a need to further explore and elucidate their therapeutic mechanisms. The bibliometric analysis also identified hotspots and trends in promoting bone regeneration by IONPs from MRI, cell labeling to *in vitro* experiments, composite scaffolds, bone tissue engineering, and hydroxyapatite. Later studies can further experiment with innovations in tissue engineering based on HA combined with IONPs, and adding other organic or inorganic material components, adding cytokines or pharmaceutical components to better accomplish the purpose of bone regeneration. It is worth mentioning that although IONPs are relatively safe for use in bone regeneration, their toxicity is influenced by some factors, and specific toxicological studies and refined interpretation protocols are areas that we will need to explore further at a later stage.
